# Gamma Rays and Sodium Azide Induced Genetic Variability in High-Yielding and Biofortified Mutant Lines in Cowpea [*Vigna unguiculata* (L.) Walp.]

**DOI:** 10.3389/fpls.2022.911049

**Published:** 2022-06-14

**Authors:** Aamir Raina, Rafiul Amin Laskar, Mohammad Rafiq Wani, Basit Latief Jan, Sajad Ali, Samiullah Khan

**Affiliations:** ^1^Mutation Breeding Laboratory, Department of Botany, Aligarh Muslim University, Aligarh, India; ^2^Botany Section, Women's College, Aligarh Muslim University, Aligarh, India; ^3^Department of Botany, Bahona College, Jorhat, India; ^4^Department of Botany, Abdul Ahad Azad Memorial Degree College Bemina, Cluster University Srinagar, Srinagar, India; ^5^Department of Clinical Pharmacy, College of Pharmacy, King Saud University, Riyadh, Saudi Arabia; ^6^Department of Biotechnology, Yeungnam University, Gyeongsan, South Korea

**Keywords:** cowpea, genetic gain, genetic variability, yield, phenotyping, selection, food security, biofortification

## Abstract

With the twin pressures of high population growth and extreme weather events, developing countries are the worst hit in meeting the food demands of their people, with millions unable to access adequate and nutritionally balanced food. Crop production must be increased by 70% to keep up with the food demands of a rapidly growing population, which is expected to rise to 9.6 billion by 2050. Legumes are ideal food crops to increase agricultural productivity and achieve sustainable development goals. Cowpea, a warm-season grain legume, is often categorized as a neglected crop with immense scope for genetic improvement through proper breeding strategies. A multi-year field experiment of induced mutagenesis was conducted to increase seed yield and genetic variability in the agro-economic traits of two cowpea varieties treated with different doses of gamma (γ) rays and sodium azide (SA). The study was also aimed to optimize different doses of γ rays and SA employed individually and in combinations. Quantitative trait analysis revealed a maximum increase in seed yield from M_2_ to M_3_ generation. Among the 10 quantitative traits studied, seeds per pod and seed weight positively correlated with a major direct impact on yield. An extensive phenotypic selection cycle from M_2_-M_4_ generations resulted in isolating new high-yielding and nutrient-dense mutant lines. Such high-yielding biofortified mutant lines with enhanced genetic variability could serve as a donor of elite genes and represent a valuable genetic resource for improving low-yielding warm-season grain legumes.

## Introduction

At present, global attention on pulses, their role in food and nutrition security, and sustainable agriculture are greater than ever. Numerous research organizations are working together at the national and international levels to solve the question on “How to feed more than nine billion people in 2050?” The United Nations Food and Agriculture Organization (FAO) 2009 emphasized the food security issues and estimated that agricultural production must increase by 70% to meet the food demands of an expected 9.6 billion people by 2050. Drastic climate change and increases in extreme weather events, such as floods, drought, heat, and salinity, pose significant risks to agriculture. Coordinated action across primary research and funding organizations is required to maximize agricultural productivity/ genetic gain (rate of increase in yield over a given period) of major pulse crops to keep up with the food demands of a continuously growing population (Varshney et al., 2018). Pulses are “Leguminosae crops harvested wholly for their grain, including beans, lentils, and cowpeas” (FAO, 1994). Among legumes, cowpea [*Vigna unguiculata* (L.) Walp.] is a self-pollinated primary pulse crop with chromosome number 2n = 2x = 22 and a genome size of 620 Mb (Arumuganathan and Earle, 1991; Herniter et al., 2019; Omirou et al., 2019). The wild cowpea plants grow only in Madagascar and tropical Africa, and hence Africa is speculated to be the center of origin (Steele, 1976). Cowpea seeds are rich in proteins, carbohydrates, and dietary minerals that fill the void of the protein deficient cereal-based human diet (Boukar et al., 2016; Samireddypalle et al., 2017). Cowpeas form a vital component of the human diet, and livestock feed, and replenish soil fertility through biological nitrogen fixation (Horn et al., 2016). Annually, the plant is cultivated on 14.4 million hectares that produce 8.90 million tons (Food and Agriculture Organization of the United Nations, 2021) of cowpea seeds. The annual average production of cowpea is low compared to other grain legumes, primarily due to narrow genetic variability and the low yielding potential of existing genotypes (Raina et al., 2020). The consistent use of conventional breeding approaches has reduced genetic variability, which is the main prerequisite for crop improvement programs (Holme et al., 2019). Therefore, new breeding techniques such as induced mutagenesis are required to accomplish the goals of increased genetic variability. Besides genetic variability, induced mutagenesis offers an opportunity to improve a single character without altering the entire genetic constitution (Shu et al., 2012; Maghuly et al., 2018). Globally, induced mutagenesis has successfully developed hundreds of agro-economically important mutant varieties (Laskar et al., 2015, 2018a; Raina et al., 2016; Khursheed et al., 2018). Up to 2022, 3,402 mutant varieties, including 468 legumes, have been developed^1^. However, only 16 cowpea mutant varieties with improved agronomic traits have been developed and officially released. In the literature body, conclusive evidence about an optimum dose of γ rays, SA, and γ rays+SA that could be used for the genetic improvement of cowpea is lacking. Instead, no combination of γ rays+SA, SA, and a variable range of γ rays has been employed in developing elite cowpea varieties. For instance, 10 cowpea mutant lines were developed by treating seeds with 100 to 300 Gy in India. In Costa Rica, a high-yielding cowpea mutant variety, viz., Uneca-Gama, was developed by treating seeds with 100 Gy γ rays. In Zimbabwe, a cowpea mutant variety, viz., CBC5 with 18% high grain and fodder yield, was developed by irradiating cowpea seeds at a dose of 150 Gy γ rays. In Zambia, a cowpea mutant variety, viz., Lunkhwakwa, with improved biotic stress tolerance and higher yield, was developed by irradiating cowpea seeds at a dose of 150 Gy γ rays (see text footnote 1). Keeping in view the production constraints of cowpea and the necessity of optimization of γ rays, SA, and γ rays+SA doses, a multi-year induced mutagenesis field experiment from April 2014 to October 2017 was undertaken to improve genetic variability and yielding potential of cowpea varieties.

## Materials and Methods

### Plant Material

Uniform and healthy seeds of two cowpea varieties, Gomati VU-89 and Pusa-578, were procured from the National Bureau of Plant Genetic Resources (NBPGR), New Delhi, India (Supplementary Table 1).

### Methodology

**Figure 1:**
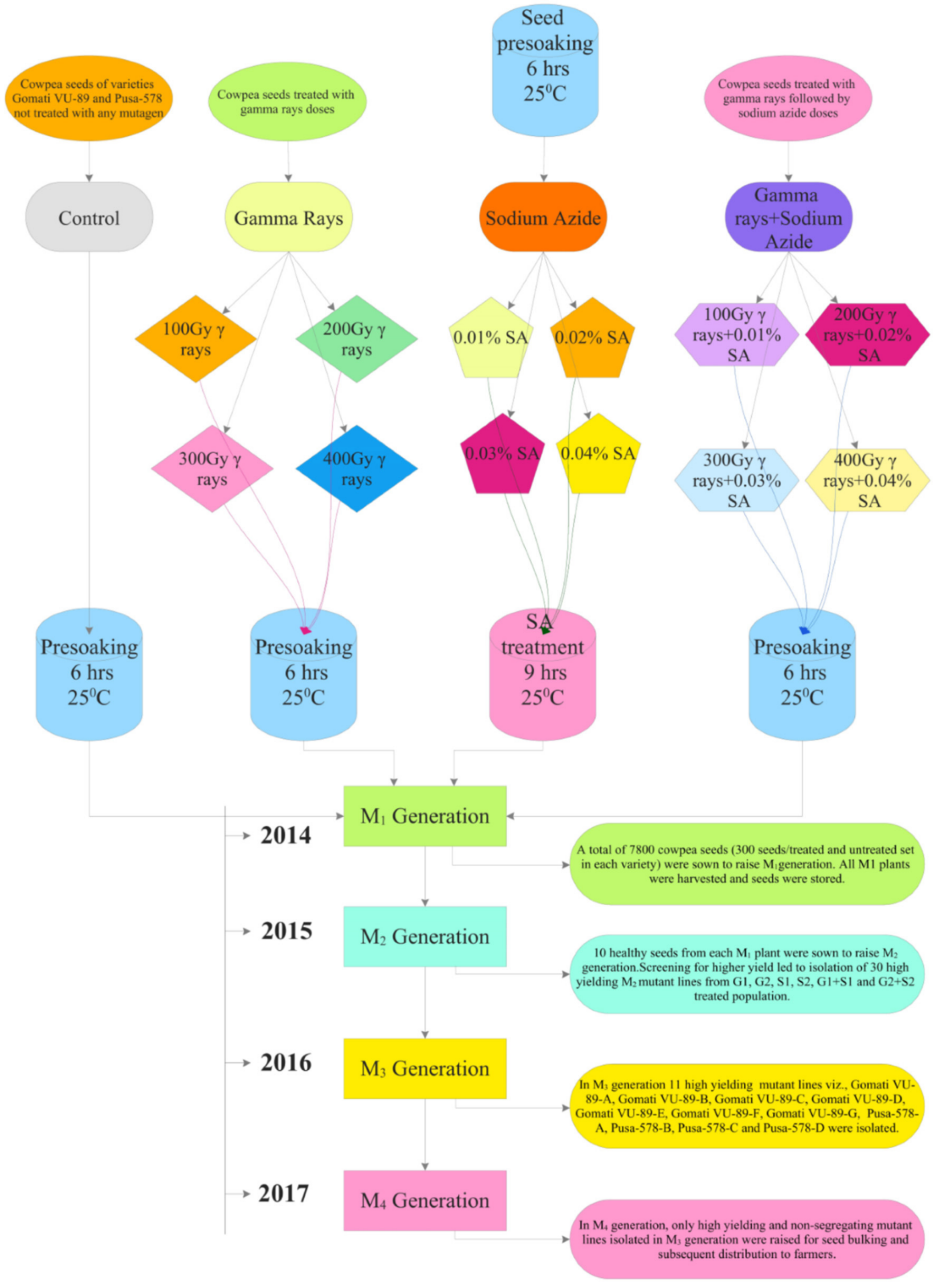
Illustration of the induced mutagenesis and selection methodology followed during 2014–2017.

At the beginning of the experimentation, we performed a radiosensitivity test to optimize the mutagen doses of γ rays and SA (see Raina et al., 2018). For γ rays treatments, we irradiated batches of 300 dry seeds with various doses viz., 100 Gy (G1), 200 Gy (G2), 300 Gy (G3), 400 Gy (G4), 500 Gy (G5), 600 Gy (G6), 700 Gy (G7), 800 Gy (G8), 900 Gy (G9), and 1,000 Gy (G10). The seeds were irradiated at a dose rate of 11.58 Gy/min using Gamma chamber Model-900 with Cobalt-60 radioisotope at National Botanical Research Institute (NBRI), Lucknow, India. We chose a minimum γ rays dose (100 Gy) following favorable responses of lentils reported by Laskar and Khan (2017). For SA treatments, an aqueous 1% (v/v) stock solution of SA, manufactured by Sissco Research Laboratories Pvt. Ltd., Mumbai, India, was prepared in phosphate buffer (pH 3.0) at Mutation Breeding Laboratory, Botany Department, AMU, Aligarh, India. From this stock solution we prepared different doses of SA such as 0.01% (S1), 0.02% (S2), 0.03% (S3), 0.04% (S4), 0.05% (S5), 0.06% (S6), 0.07% (S7), 0.08% (S8), 0.09% (S9), and 0.1% (S10). Furthermore, we chose a minimum SA dose (0.01%) following the favorable growth responses of linseed reported by Alka and Ansari (2013). Before SA treatments, batches of 300 dry and healthy seeds per treatment were placed in mesh bags for pre-soaking in double-distilled water for 6 h and then treated with S1-S10 SA doses for 9 h. For combined mutagen treatments, batches of 300 γ irradiated seeds were treated with respective SA doses and denoted as S1+G1 (100 Gy γ rays+0.01% SA), S2+G2 (200 Gy γ rays+0.02% SA), S3+G3 (300 Gy γ rays+0.03% SA) S4+G4 (400 Gy γ rays+0.04% SA), S5+G5 (500 Gy γ rays+0.05% SA), S6+G6 (600 Gy γ rays+0.06% SA), S7+G7 (700 Gy γ rays+0.07% SA), S8+G8 (800 Gy γ rays+0.08% SA), S9+G9 (900 Gy γ rays+0.09% SA), and S10+G10 (1000 Gy γ rays+0.1% SA). Doses beyond 400 Gy in γ rays, 0.04% SA in SA, and 400 Gy γ rays+0.04% SA in combination treatments caused more than a 50% reduction in seed germination. Hence, we did not include such doses for study in subsequent generations (Supplementary Figures 1–3). The selection procedure followed during the experimentation is illustrated in Figure 1.

#### Details of the 2014–2017 Field Trials

##### Year 1, 2014

During mid-April 2014, a total of 7,800 cowpea seeds (300/treatment and control set/variety) were sown in a randomized complete block design (RCBD) with 30 replications in 23.5 × 40 m sized agriculture farm of AMU, Aligarh (Supplementary Table 2; Supplementary Figure 4). Seeds were sown at a distance of 60 cm inter-row and 30 cm intra-row spacing to raise the M_1_ generation. All recommended agricultural practices such as regular irrigation, fertilizers as a source of nitrogen (N), phosphorus (P), and potassium (K) at a rate of 20 kg N ha^−1^, 35 kg P_2_O_5_ ha^−1^, and 20 kg K_2_O ha^−1^, respectively, were added. During mid-October 2014, all the seeds from M_1_ plants were harvested separately and stored for raising subsequent generations.

##### Year 2, 2015

During mid-April 2015, 10 healthy seeds from each M_1_ plant were sown to raise M_2_ generation. A total of 57,620 M_2_ seeds generated from the M_1_ generation of two varieties were sown in the same field to raise M_2_ generation. Moreover, 47,650 seeds germinated, of which 38,749 plants survived were screened for morphological diversity (Supplementary Tables 2, 3). The mean data from 30 replicates on 10 quantitative phenotypic traits included plant height (PH), days to flowering (DF), days to maturity (DM), pods per plant (PPP), seeds per pod (SPP), seed weight (SW), branches per plant (BPP), pod length (PL), plant yield (PY), and harvest index (HI) (Supplementary Table 4). At maturity, plants were harvested manually and sun-dried for 7 days to determine the dry weight of aboveground biomass. The harvest index was calculated by taking the ratio of plant yield to biological yield (dry weight of aboveground biomass). Selection for high yielding mutant lines was carried out in both varieties. Based on the quantitative statistics, 30 high yielding M_2_ mutant lines were isolated from each G1, G2, S1, S2, G1+S1, and G2+S2 treated population and advanced to subsequent M_3_ generation. In mid-October 2015, all the seeds from high yielding mutant lines were harvested separately and stored for raising subsequent generations.

##### Year 3, 2016

**Figure 2:**
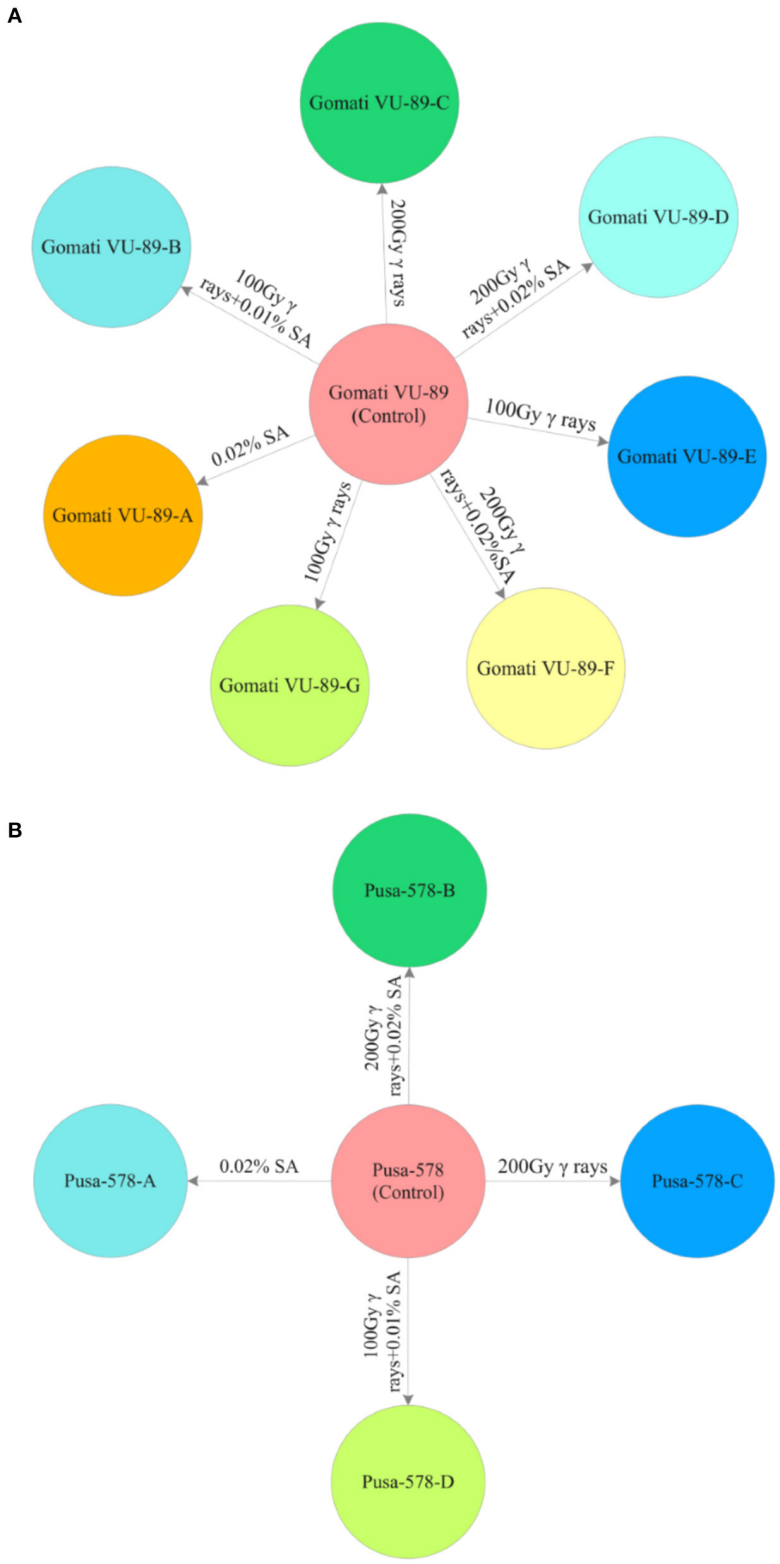
M_3_ high yielding mutant lines isolated in different mutagenic treatments in **(A)** variety Gomati VU-89 and **(B)** variety Pusa-578.

From the M_2_ generation onwards, we advanced only high yielding and non-segregating mutant lines to M_3_ and M_4_ generations. In mid-April 2016, 10 healthy seeds from high yielding M_2_ mutant lines were sown to raise the M_3_ generation. A total of 3,600 M_2_ seeds generated from M_2_ high yielding mutant lines were sown in the same field to raise M_3_ generation (Supplementary Tables 2, 3). The effective selection process led to the isolation of 11 high yielding and non-segregating mutant lines viz., Gomati VU-89-A, Gomati VU-89-B, Gomati VU-89-C, Gomati VU-89-D, Gomati VU-89-E, Gomati VU-89-F, Gomati VU-89-G, Pusa-578-A, Pusa-578-B, Pusa-578-C, and Pusa-578-D in lower and medium γ rays and SA treated populations (Figure 2). During mid-October 2016, all the seeds from 11 high yielding mutant lines were harvested separately and stored for raising subsequent generations.

##### Year 4, 2017

**Figure 3:**
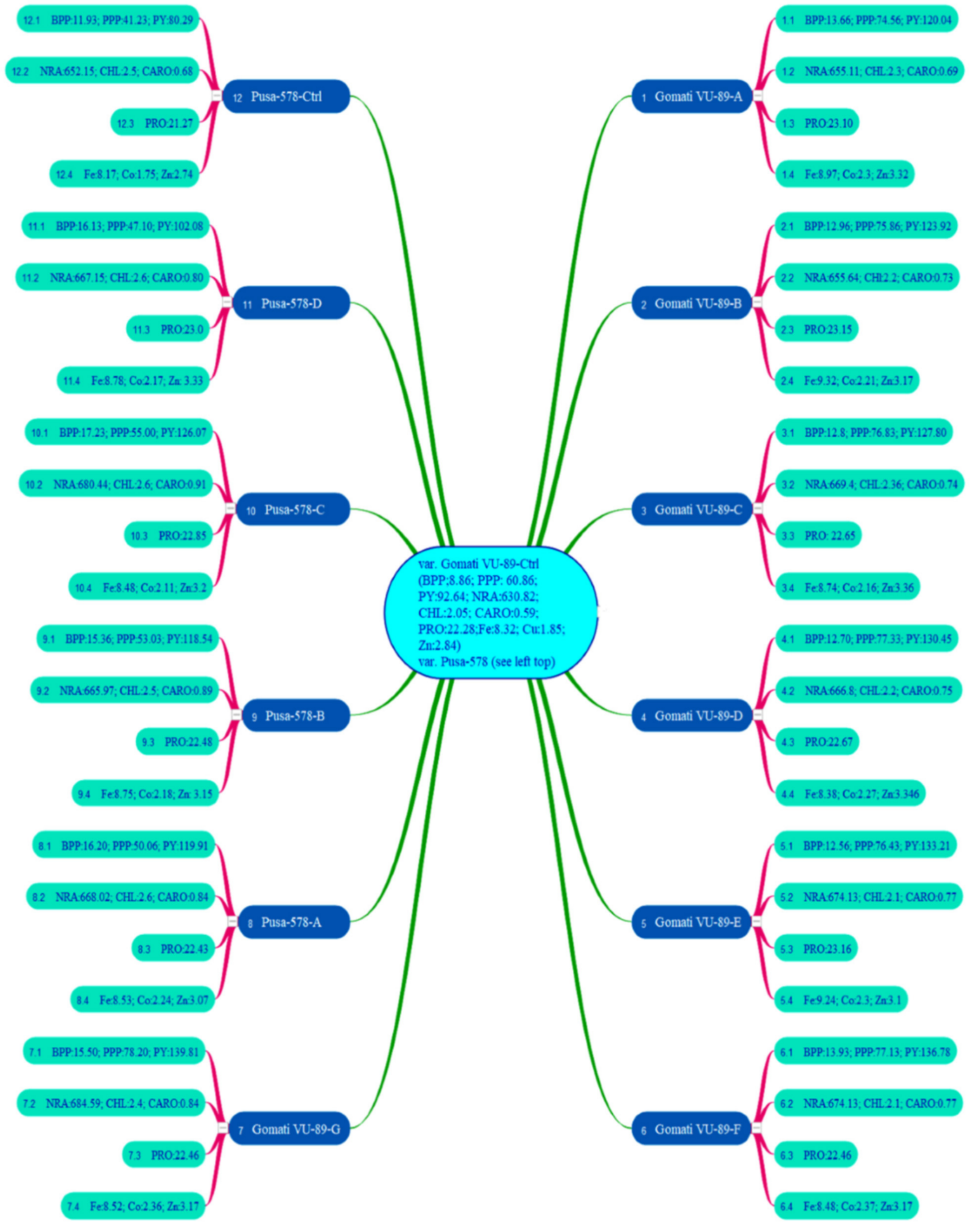
Mean values of branches per plant (BPP), pods per plant (PPP), plant yield (PY), nitrate reductase activity (NRA), chlorophyll (CHL), carotenoids (CARO), proteins (PRO), iron (Fe), copper (Cu), zinc (Zn) in 11 M_4_ high yielding mutant lines and two control plants (*n* = 30). Measurement unit for NRA (nmol h^−1^ g^−1^ FW); CHL (mg g^−1^ FW); CARO (mg g^−1^ FW); PRO (%); Fe (mg 100 g^−1^); Co (mg 100 g^−1^); Zn (mg 100 g^−1^) (Source: Data adapted from Raina et al., 2020. http://creativecommons.org/licenses/by/4.0/).

In the M_4_ generation, we raised only 11 high yielding and non-segregating mutant lines during mid-April 2017. The data was recorded on quantitative, physiological, and biochemical traits to develop a relative profile of each mutant line (see Raina et al., 2020). In addition, we assessed M_4_ high yielding mutant lines for seed micronutrients (mg. 100 g^−1^) such as iron (Fe), copper (Cu), and zinc (Zn) using atomic absorption spectrophotometry. We also evaluated such mutant lines for nitrate reductase activity (nmol NO_2_.g^−1^.h^−1^FW), chlorophyll, and carotenoid (mg.g^−1^ fresh leaf mass) contents following the method of MacKinney (1941) and Jaworski (1971), respectively (see Figure 3). The chlorophyll and carotenoid contents were measured as per the equation of Arnon (1949).


$$\begin{array}{lll} \text{Chlorophyll} & = & \;\left\{20.2\left({\text{OD}}_{645}\right)\;+\;8.02\left({\text{OD}}_{663}\right)\right\}\;\times \;\frac{\text{V}}{1000\;\times \text{ W}}\\ \text{Carotenoid} & = & \;\frac{7.6\;\left({\text{OD}}_{480}\right)-\;1.49\;\left({\text{OD}}_{510}\right)}{\text{d x }1000\text{ x W}}\times \text{ V} \end{array}$$

where

OD_645_, OD_663_, OD_480_, OD_510_ = Optical densities at respective wavelength

V = Volume of an extract; W = Mass of leaf tissues; d = Length of light path (1.4 cm).

### Statistical Analysis

One-way analysis of variance (ANOVA) followed by Duncan's multiple range *post hoc* test (DMRT) was performed to evaluate the significance of the quantitative phenotypic data (*p* ≤ 0.05) (R Core Team, 2019). The Spearman's correlation coefficients were computed to visualize the relationship between different phenotypic quantitative traits using Performance Analytics (https://CRAN.Rproject.org/package=PerformanceAnalytics) and Hmisc (https://CRAN.R-project.org/package=Hmisc) R packages. Path coefficients were calculated to examine the direct and indirect impact of quantitative phenotypic characters on total plant yield using SPSS AMOS version 26. Hierarchical Cluster Analysis (HCA) was performed using SPSS version 16.0 (Team EQX) to evaluate genetic distance and degree of heterogeneity. In addition, data were also subjected to Principal Component Analysis (PCA) to assess the similarities between the quantitative phenotypic traits using the FactoMineR (https://CRAN.R-project.org/package=FactoMineR) and factoextra (https://CRAN.R-project.org/package=factoextra) packages. The interpopulation proximity matrix based on Euclidean distance was performed using SPSS version 16.0. The genetic parameters were calculated following the formula proposed by Allard (1960).


$$\text{GCV }\left(\%\right)=\frac{\sqrt{\sigma^{\text{2}}\text{g}}}{\bar{\text{x}}}\times \text{ 100}$$

where GCV, Genotypic coefficient of variation; σ^2^ g (genotypic variance); (MSG –MSE)/r; MSG is a measure of the average square of tested accession; MSE is a measure of the average square of error; r is a number of replications.


$${\text{h}}^{2}(\%)=\frac{\sigma^{\text{2}}\text{g}}{\sigma^{\text{2}}\text{p}}\;\times \text{ 100}$$

where h^2^ refers to heritability and σ^2^ p refers to phenotypic variance


$$\begin{array}{l} \text{GA }\left(\text{\% of mean}\right)=\text{k}.\sigma \text{p}.{\text{h}}^{2} \end{array}$$

where GA refers to genetic advance, σp refers to phenotypic standard deviation of the average performance of the treated population; K = 2.64, constant for 1% selection intensity.

## Results

**Figure 4:**
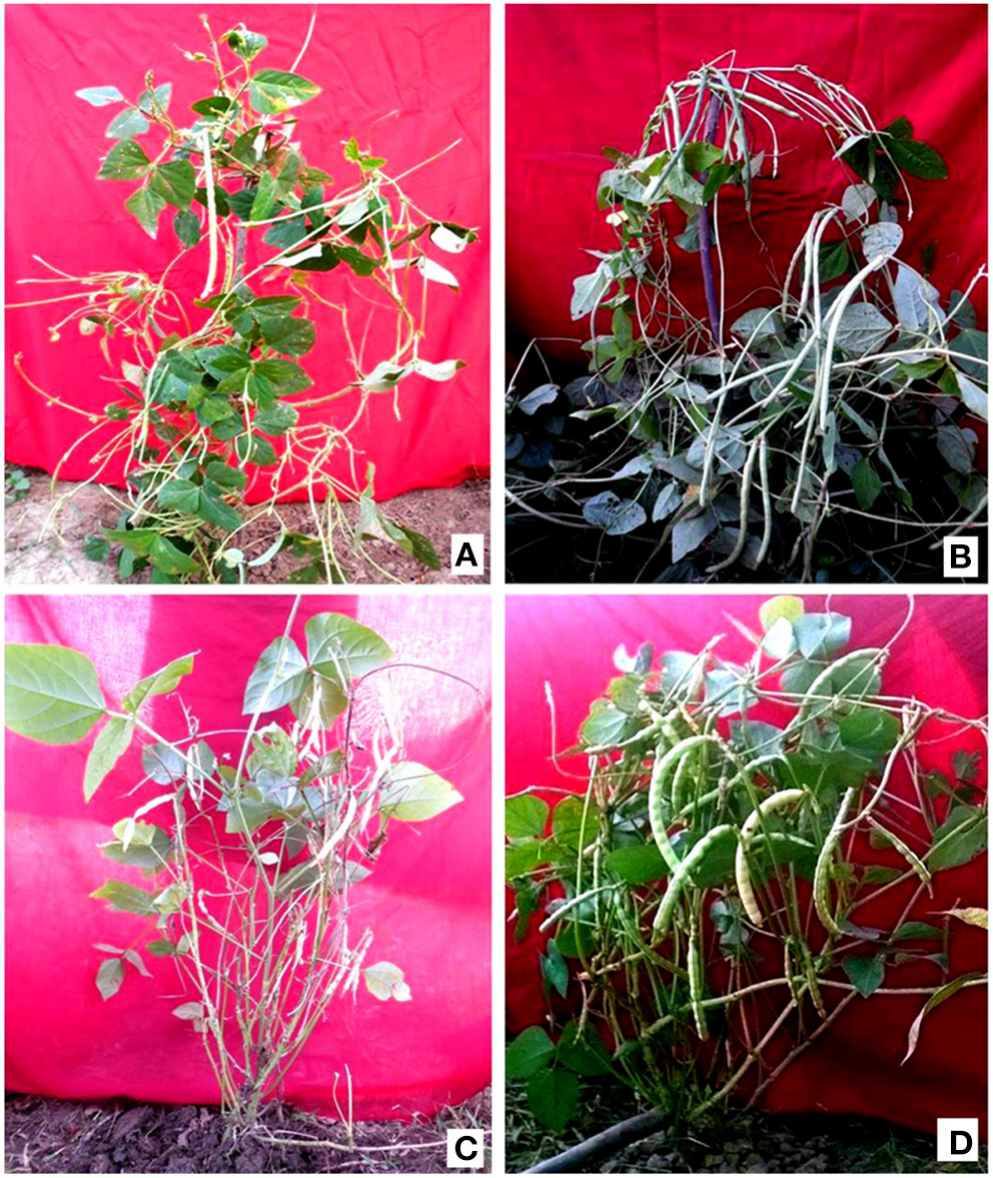
**(A)** Control plant of variety Gomati VU-89; **(B)** High yielding mutant of variety Gomati VU-89; **(C)** Control plant of variety Pusa-578; **(D)** High yielding mutant of variety Pusa-578.

**Figure 5:**
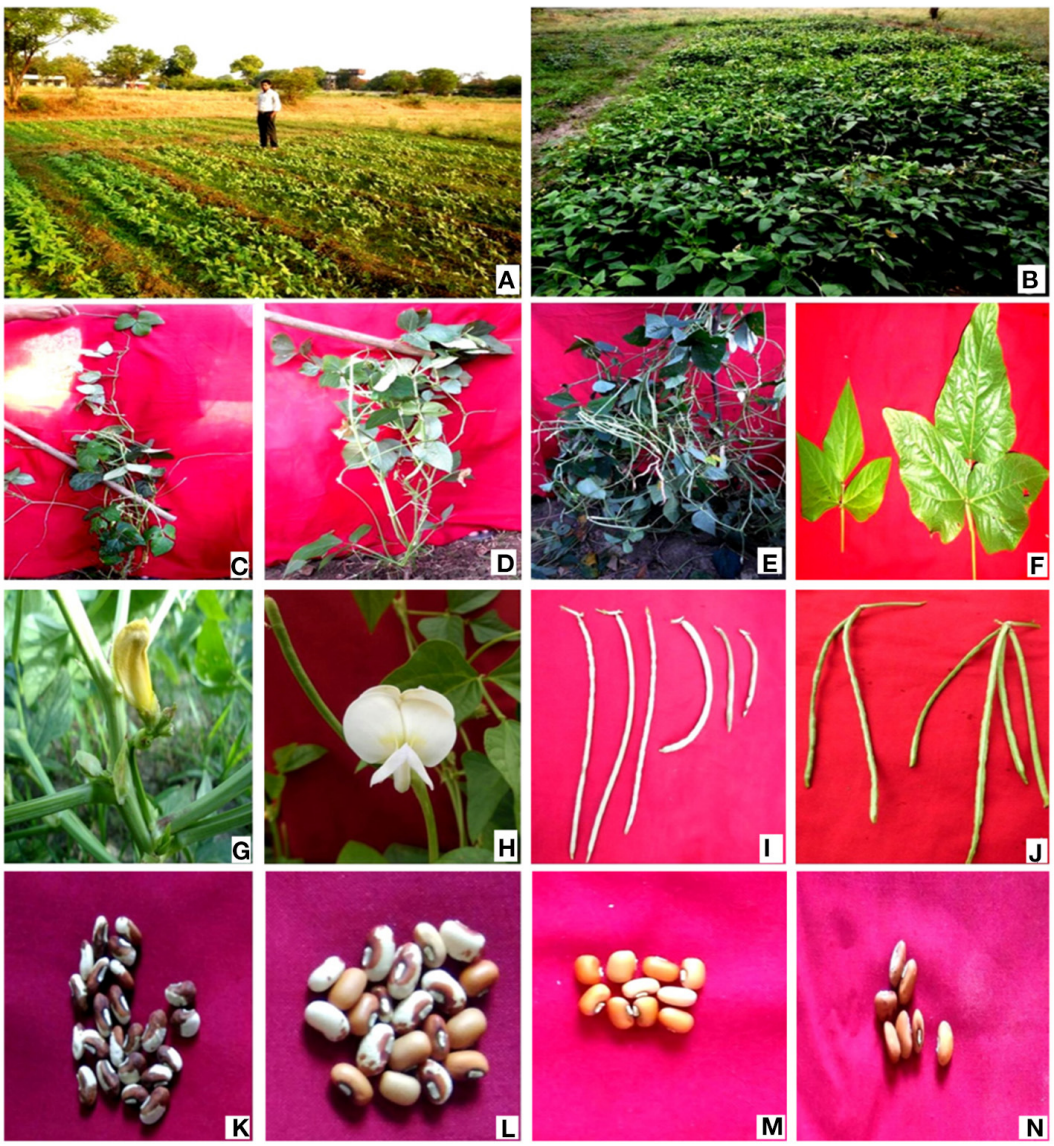
**(A)** Experimental field (seedling stage); **(B)** Experimental field (harvesting stage); **(C)** Tall mutant; **(D)** Semi-dwarf mutant; **(E)** Bushy mutant; **(F)** variation in leaf size; **(G,H)** variation in flower color; **(I)** variation in pod length; **(J)** variation in pod number; **(K,L)** variation in seed size; **(M,N)** variation in seed coat.

The different doses of γ rays and SA induced substantial variations in the morphological traits, resulting in the selection of eleven high yielding and non-segregating mutant lines (Figures 4, 5). Such variations in morphological traits represent a valuable genetic resource for future cowpea breeding programs. The estimation of genetic gain in the M_2_ generation facilitated the selection of mutagen treated population for advancement into the subsequent M_3_ generation. The results revealed a substantial improvement in a genetic gain from M_2_ to M_3_ generation with the highest increase in G1 treated populations in Gomati VU-89 (9.28%) and Pusa-578 (14.15%) varieties. However, G1 and S1 treatments showed a maximum increase in genetic gain in the varieties Gomati VU-89 (28.75%) and Pusa-578 (45.73%), respectively, in M_3_ generation (Supplementary Table 5).

### Quantitative Genetic Parameters: Effect of Mutagen Doses on Quantitative and Genetic Parameters

In both M_2_ and M_3_ generations, mutagen induced genetic variability in yield and yield attributing traits were evaluated based on descriptive statistics. One-way ANOVA revealed a significant increase in mean values for all the quantitative traits except seeds per pod in both the varieties and seed weight in variety Pusa-578 (Supplementary Table 6). The results also revealed a high genetic variability coupled with high heritability in both generations. In addition, lower and medium doses of γ rays and SA were more effective in increasing the mean values of quantitative traits as mentioned below:

#### Plant Height (cm)

In M_2_ generation, mutagen doses significantly reduced the plant height with the maximum decrease in G4+S4 treatment in the varieties Gomati VU-89 (174.36 cm) and Pusa-578 (173.30 cm) (Tables 1, 2). The maximum GCV (1.96%; 4.33%) and GA (2.42%; 10.82%) were recorded in S2 treatment in the varieties Gomati VU-89 and Pusa-578, respectively. The highest h^2^ was recorded in G2 and S2 treatments in Gomati VU-89 (76.42%) and Pusa-578 (89.59%), respectively.

**Table 1:** Estimates of mean values ± standard error (*n* = 30), genotypic coefficient of variation (GCV %), heritability (h^2^%), and genetic advance as % of the mean (GA %) for 10 quantitative traits in M_2_ generation of cowpea variety Gomati VU-89.

**Characters**		**Treatment population**												
		**C**	**G1**	**G2**	**G3**	**G4**	**S1**	**S2**	**S3**	**S4**	**G1+S1**	**G2+S2**	**G3+S3**	**G4+S4**
Plant height	MEAN	182.61^a^ ± 0.2	179.92^b^ ± 0.41	178.82^c^ ± 0.34	178.31^cd^ ± 0.28	176.72^ef^ ± 0.2	181.79^a^ ± 0.35	179.19^bc^ ± 0.51	177.57^de^ ± 0.28	174.41^g^ ± 0.26	178.87^c^ ± 0.34	177.63^de^ ± 0.31	175.89^f^ ± 0.25	174.36^g^ ± 0.26
	GCV%	0.32	1.4	1.07	0.92	0.75	1.21	1.96	0.92	1.08	1.23	1.12	0.70	0.84
	h^2^%	22.22	63.56	76.42	61.64	66.66	64.37	70.10	60.40	53.08	66.03	66.72	49.51	53.31
	GA	0.21	1.64	1.58	1.07	0.92	1.41	2.42	1.06	0.47	1.47	1.36	0.74	0.96
Days to flowering	MEAN	80.16^a^ ± 0.32	77.46^c^ ± 0.37	77.36^c^ ± 0.30	77.86^c^ ± 0.33	77.66^c^ ± 0.38	77.56^c^ ± 0.37	77.76^c^ ± 0.38	78.00^c^ ± 0.33	78.26^bc^ ± 0.26	77.96^c^ ± 0.35	77.80^c^ ± 0.39	79.10^b^ ± 0.38	78.53^bc^ ± 0.35
	GCV%	1.78	3.77	2.54	2.95	2.27	3.64	3.70	2.84	2.18	3.55	3.95	2.44	2.06
	h^2^%	44.15	78.05	67.60	70.72	45.90	77.04	75.40	69.00	66.49	78.57	78.52	51.79	45.14
	GA	3.13	8.80	5.52	6.55	4.06	8.44	8.50	6.24	4.70	8.32	9.26	4.64	3.65
Days to maturity	MEAN	154.83^a^ ± 0.57	150.16^bc^ ± 0.59	150.26^bc^ ± 0.57	150.46^b^ ± 0.50	149.80^bc^ ± 0.58	151.20^b^ ± 0.81	149.03^c^ ± 0.69	150.26^bc^ ± 0.62	149.36^bc^ ± 0.49	149.73^bc^ ± 0.68	148.86^c^ ± 0.66	145.00^d^ ± 0.62	143.10^e^ ± 0.55
	GCV%	1.04	3.34	2.94	2.30	2.47	3.83	3.68	2.71	2.07	4.12	4.02	2.50	2.05
	h^2^%	22.50	82.49	77.37	70.73	65.81	72.50	78.83	67.76	65.14	86.08	86.84	60.52	54.59
	GA	1.30	8.02	6.84	5.11	5.30	8.62	8.64	5.90	4.41	10.10	9.91	5.13	4.00
Pods per plant	MEAN	60.10^fg^ ± 0.55	66.46^ab^ ± 0.93	64.20^bcd^ ± 0.85	62.53^de^ ± 0.67	61.00^ef^ ± 0.55	63.10^cde^ ± 0.86	65.53^b^ ± 0.82	65.30^b^ ± 0.67	59.43^fg^ ± 0.49	64.50^bcd^ ± 0.90	68.03^a^ ± 0.72	58.03^gh^ ± 0.17	56.96^h^ ± 0.54
	GCV%	4.13	13.64	12.10	7.89	6.25	11.46	11.76	7.40	6.26	12.52	10.18	8.66	5.51
	h^2^%	44.28	90.39	87.18	74.74	70.99	82.26	88.20	72.95	75.89	84.99	89.84	71.86	60.21
	GA	7.27	34.24	29.83	18.02	13.92	27.45	29.18	16.69	14.41	30.49	25.48	19.39	11.30
Branches per plant	MEAN	8.63^g^ ± 0.18	11.50^b^ ± 0.26	10.00^d^ ± 0.23	9.13^efg^ ± 0.19	9.23^efg^ ± 0.20	10.66^c^ ± 0.21	9.50^def^ ± 0.20	9.03^fg^ ± 0.15	8.80 ^g^ ± 0.16	10.00^d^ ± 0.19	12.46 ^a^ ± 0.28	9.03f ^g^ ± 0.18	9.76d^e^ ± 0.21
	GCV%	8.52	15.95	17.59	14.06	12.56	11.17	16.84	12.87	11.03	14.47	18.16	11.17	11.12
	h^2^%	38.74	72.01	74.71	67.73	57.97	57.00	77.61	75.92	62.98	74.57	79.06	57.20	50.80
	GA	14.01	35.75	40.14	30.55	25.26	22.27	39.16	29.60	22.98	33.00	42.62	22.30	20.93
Seeds per pod	MEAN	12.00^b^ ± 0.12	12.50^ab^ ± 0.17	12.40^ab^ ± 0.16	12.30^ab^ ± 0.16	12.10^ab^ ± 0.13	12.80^a^ ± 0.18	12.40^ab^ ± 0.22	12.26^ab^ ± 0.19	12.23^ab^ ± 0.16	12.20^ab^ ± 0.26	12.03^b^ ± 0.25	12.00^b^ ± 0.18	11.96^b^ ± 0.18
	GCV%	4.63	9.44	9.42	8.04	6.10	11.15	14.53	10.75	8.79	17.46	14.32	12.19	9.36
	h^2^%	43.33	70.90	73.21	64.11	58.86	76.70	79.11	70.89	67.86	80.21	70.58	78.94	63.75
	GA	8.05	20.96	21.29	17.00	12.36	25.78	34.12	23.90	19.11	41.28	31.76	28.61	19.74
Seed weight	MEAN	13.00^b^ ± 0.17	13.50^a^ ± 0.15	13.40^ab^ ± 0.19	13.30^ab^ ± 0.16	13.20^ab^ ± 0.17	13.70^a^ ± 0.14	13.40^ab^ ± 0.13	13.20^ab^ ± 0.13	13.20^ab^ ± 0.13	13.45^a^ ± 0.20	13.06^b^ ± 0.22	13.00^b^ ± 0.18	12.90^b^ ± 0.19
	GCV%	6.34	8.30	10.26	8.81	7.16	8.01	7.54	6.99	6.36	11.43	14.51	9.03	8.23
	h^2^%	47.07	75.06	73.15	67.50	56.69	76.27	76.70	72.18	64.50	73.90	79.75	64.96	57.88
	GA	11.49	19.00	23.17	17.76	14.24	18.47	17.45	15.69	13.50	25.94	34.21	19.21	16.54
Pod length	MEAN	29.57^g^ ± 0.14	30.73^def^ ± 0.10	31.02^cde^ ± 0.16	31.12^bcd^ ± 0.08	30.58^ef^ ± 0.13	31.29^bc^ ± 0.16	31.54^b^ ± 0.13	30.43^f^ ± 0.08	29.63^g^ ± 0.14	31.06^bcde^ ± 0.20	32.03^a^ ± 0.24	29.58^g^ ± 0.19	29.17^g^ ± 0.24
	GCV%	0.95	2.79	3.85	2.09	1.87	4.26	3.3	1.93	1.88	4.71	4.52	3.37	2.59
	h^2^%	12.69	75.67	75.66	79.24	42.66	81.27	79.41	74.76	38.02	71.97	61.73	52.39	26.61
	GA	1.51	18.19	28.52	15.72	10.42	32.42	24.57	4.41	3.06	10.55	9.39	6.44	3.53
Plant yield	MEAN	93.75^h^ ± 0.32	112.00^a^ ± 0.99	106.67^cd^ ± 0.78	102.28^f^ ± 060	97.42^g^ ± 0.48	110.01^a^ ± 1.03	108.00^bc^ ± 0.78	105.50^de^ ± 0.62	95.94^g^ ± 0.52	103.80^ef^ ± 0.87	106.97^cd^ ± 081	90.52^i^ ± 0.77	87.80^j^ ± 0.72
	GCV%	1.49	7.43	5.64	4.02	3.32	7.15	5.45	4.19	3.55	5.86	5.37	4.27	3.94
	h^2^%	41.59	82.46	77.65	70.05	69.30	77.04	76.00	72.31	66.84	71.65	72.16	50.81	48.53
	GA	2.54	17.81	13.12	8.90	7.30	16.58	12.56	9.41	7.67	13.11	12.06	8.05	7.25
Harvest index	MEAN	27.13^j^ ± 0.22	41.25^a^ ± 0.52	36.62^bc^ ± 0.44	38.31^d^ ± 0.39	36.37^ef^ ± 0.34	40.66^ab^ ± 0.47	39.02^cd^ ± 0.38	36.65^ef^ ± 0.35	34.87^g^ ± 0.43	37.13^e^ ± 0.46	35.90^fg^ ± 0.37	32.52^h^ ± 0.34	30.05^i^ ± 0.26
	GCV%	3.42	9.35	18.17	7.17	5.82	9.93	8.21	6.92	6.48	7.92	6.56	6.05	5.22
	h^2^%	41.15	74.87	73.19	70.77	64.14	83.23	81.43	73.24	53.60	66.13	64.81	58.82	62.25
	GA	5.79	21.36	18.17	11.18	12.32	23.93	19.56	15.65	12.53	17.01	13.95	12.26	10.89

*Means followed by the same letter is not different at a 5% level of significance, based on the DMRT*.

**Table 2:** Estimates of mean values ± standard error (*n* = 30), genotypic coefficient of variation (GCV %), heritability (h^2^%), and genetic advance as % of the mean (GA %) for 10 quantitative traits in M_2_ generation of cowpea variety Pusa-578.

**Characters**		**Treatment population**												
		**C**	**G1**	**G2**	**G3**	**G4**	**S1**	**S2**	**S3**	**S4**	**G1+S1**	**G2+S2**	**G3+S3**	**G4+S4**
Plant height	MEAN	180.32^a^ ± 0.29	178.51^ab^ ± 0.56	177.51^b^ ± 0.58	177.00^d^ ± 0.50	176.80^d^ ± 0.49	179.30^ab^ ± 0.79	178.10^bc^ ± 0.80	177.70^bc^ ± 0.71	177.30^bc^ ± 0.58	177.31^bc^ ± 0.72	175.34^de^ ± 062	174.50^ef^ ± 0.64	173.30^f^ ± 0.62
	GCV%	0.79	2.90	3.10	2.09	1.75	4.14	4.33	3.12	2.44	3.80	3.50	2.81	2.60
	h^2^%	48.48	87.02	88.44	74.05	64.66	88.56	89.59	77.67	75.52	88.00	85.62	76.94	73.81
	GA	1.46	7.15	7.72	4.74	3.72	10.29	10.82	7.26	5.61	9.41	8.56	6.51	5.90
Days to flowering	MEAN	87.46^a^ ± 0.40	84.60^b^ ± 0.61	85.40^b^ ± 0.60	85.3^b^ ± 0.55	85.10^b^ ± 0.42	84.63^b^ ± 0.62	86.23^ab^ ± 0.55	85.20^b^ ± 0.59	84.80^b^ ± 0.55	85.30^b^ ± 0.64	84.90^b^ ± 0.59	85.03^b^ ± 0.62	84.70^b^ ± 0.49
	GCV%	2.45	6.03	6.20	4.61	4.05	6.10	5.20	4.80	4.38	6.21	5.32	4.86	3.42
	h^2^%	53.94	82.08	78.34	72.44	80.41	81.78	80.75	70.85	69.10	81.33	76.57	68.43	60.79
	GA	4.75	14.43	12.85	10.38	9.60	14.58	12.35	10.66	9.63	14.80	12.31	10.61	7.06
Days to maturity	MEAN	160.10^a^ ± 0.44	154.00^bcd^ ± 0.59	154.30^bc^ ± 0.66	154.20^bcd^ ± 0.64	152.93^cd^ ± 0.58	156.00^b^ ± 0.93	156.00^b^ ± 0.80	155.40^b^ ± 0.67	155.00^bc^ ± 0.69	154.13^bcd^ ± 0.63	154.90^bc^ ± 0.83	152.13^d^ ± 0.58	150.10^e^ ± 0.53
	GCV%	0.99	2.87	3.36	2.89	2.54	4.77	4.09	3.17	2.77	3.09	4.27	2.98	2.49
	h^2^%	33.00	75.69	78.18	71.40	68.38	79.49	79.29	74.49	64.34	76.64	79.62	77.65	72.02
	GA	1.50	6.59	7.84	6.45	5.56	11.25	9.63	7.49	5.87	7.16	10.07	6.94	5.57
Pods per plant	MEAN	41.90^de^ ± 0.27	45.00^b^ ± 0.51	48.56^a^ ± 0.62	44.16^bc^ ± 0.41	41.16^e^ ± 0.36	44.90^b^ ± 0.46	43.80^bc^ ± 0.57	44.66^bc^ ± 0.41	41.03^e^ ± 0.39	43.90^bc^ ± 0.59	43.26^cd^ ± 0.54	40.63^e^ ± 0.59	38.26^f^ ± 0.49
	GCV%	3.25	9.13	10.60	6.60	5.64	7.99	10.16	7.35	6.28	10.59	9.46	8.28	5.42
	h^2^%	50.94	79.77	81.38	72.42	65.43	77.61	77.98	78.67	67.78	78.74	75.94	58.10	40.50
	GA	6.12	21.54	25.26	14.83	12.06	18.58	23.67	17.22	13.67	24.81	21.77	16.69	9.11
Branches per plant	MEAN	11.50^efg^ ± 0.21	12.43^d^ ± 0.24	15.03^a^ ± 0.30	11.83^def^ ± 0.20	11.23^fg^ ± 0.18	13.70^c^ ± 0.25	13.23^c^ ± 0.23	12.10^de^ ± 0.19	11.53^efg^ ± 0.19	14.36^b^ ± 0.25	13.10^c^ ± 0.28	11.73^defg^ ± 0.18	11.10^g^ ± 0.18
	GCV%	7.99	13.51	15.99	12.17	11.03	15.44	13.11	11.25	10.80	15.70	16.40	10.92	10.75
	h^2^%	41.04	69.92	79.65	70.77	67.56	81.04	75.53	70.24	64.79	85.24	75.47	71.15	65.89
	GA	13.52	29.83	37.68	27.03	23.95	36.71	30.09	24.90	23.49	38.27	37.61	24.33	23.05
Seeds per pod	MEAN	9.50^def^ ± 0.10	10.66^ab^ ± 0.15	10.20^bc^ ± 0.12	10.00^bcde^ ± 0.09	9.96^bcde^ ± 0.10	11.00^a^ ± 0.17	10.10^bcd^ ± 0.14	9.80^cdef^ ± 0.13	9.40^f^ ± 0.11	10.30^ab^ ± 0.16	9.70^def^ ± 0.15	9.60^def^ ± 0.10	9.60^def^ ± 0.11
	GCV%	5.50	10.19	8.23	7.07	6.52	12.70	11.42	10.86	8.74	11.22	10.81	7.07	5.88
	h^2^%	51.11	72.33	71.15	75.00	66.37	80.39	78.84	77.27	76.95	73.71	71.25	68.31	50.75
	GA	10.39	22.89	18.32	16.16	14.03	30.06	26.77	25.21	19.86	25.43	24.32	15.44	11.06
Seed weight	MEAN	21.01^d^ ± 0.20	22.15^a^ ± 0.27	21.58^abcd^ ± 0.31	21.10^cd^ ± 0.25	21.01^d^ ± 0.29	22.40^a^ ± 0.29	22.10^ab^ ± 0.30	21.50^abcd^ ± 0.24	21.00^d^ ± 0.22	22.20^a^ ± 0.36	21.40^bcd^ ± 0.34	21.35^bcd^ ± 032	20.95^d^ ± 0.31
	GCV%	4.63	9.86	13.43	8.13	7.33	9.40	9.03	7.24	6.46	12.12	12.81	8.92	8.53
	h^2^%	49.14	78.27	82.22	69.07	52.97	72.86	67.22	65.17	61.91	74.66	78.77	60.27	59.65
	GA	8.57	23.04	32.17	17.85	14.09	21.19	19.56	15.44	13.42	27.66	30.02	18.29	17.39
Pod length	MEAN	23.52^e^ ± 0.26	24.48^d^ ± 0.17	27.72^a^ ± 0.29	24.28^d^ ± 0.17	24.09^de^ ± 0.16	26.72^b^ ± 0.29	25.88^c^ ± 0.22	24.40^d^ ± 0.21	24.22^d^ ± 0.17	26.21^bc^ ± 0.26	26.09^bc^ ± 0.25	24.61^d^ ± 0.15	24.08^de^ ± 0.14
	GCV%	2.78	4.39	8.41	4.21	3.26	7.52	6.20	4.80	3.94	7.58	6.18	3.95	3.30
	h^2^%	18.51	62.31	79.80	59.94	49.22	69.23	72.52	56.73	57.82	77.08	65.57	64.83	57.62
	GA	3.16	9.16	19.85	8.61	6.04	16.52	13.94	9.54	7.91	17.59	13.21	8.40	6.62
Plant yield	MEAN	83.51^li^ ± 0.25	105.54^b^ ± 0.58	101.90^c^ ± 0.40	93.17^f^ ± 0.40	86.09^h^ ± 0.44	110.63^a^ ± 0.68	97.76^e^ ± 0.43	94.09^j^ ± 0.40	81.01^j^ ± 0.35	100.38^d^ ± 0.72	89.37^g^ ± 0.62	83.29^i^ ± 0.51	78.39^k^ ± 0.40
	GCV%	1.11	5.01	3.62	3.33	3.02	5.93	3.64	3.00	2.85	6.35	5.57	4.24	3.17
	h^2^%	34.02	86.26	87.22	77.10	60.86	89.29	81.33	72.35	67.41	91.52	79.65	70.98	64.11
	GA	1.72	12.28	8.94	7.72	6.23	14.80	8.68	6.75	6.18	16.50	13.13	9.44	6.71
Harvest index	MEAN	28.93^h^ ± 0.31	38.51^a^ ± 0.39	35.32^bc^ ± 0.33	34.30^cd^ ± 0.38	32.66^f^ ± 0.39	37.54^a^ ± 0.45	30.53^g^ ± 0.28	34.89^bcd^ ± 0.33	33.16^ef^ ± 0.30	35.58^b^ ± 0.40	34.00^de^ ± 0.35	29.41^h^ ± 0.30	27.74^i^ ± 0.29
	GCV%	2.71	7.18	5.16	6.09	5.66	7.73	4.83	4.49	4.01	7.06	6.39	4.98	4.23
	h^2^%	18.78	71.25	56.06	56.07	46.55	71.90	52.09	46.94	42.94	64.67	63.95	49.32	38.74
	GA	3.10	16.01	10.21	12.04	10.21	17.32	9.20	8.13	6.95	15.00	13.50	9.24	6.95

*Means followed by the same letter is not different at a 5% level of significance, based on the DMRT*.

In M_3_ generation, all the mutagen treatments significantly reduced the plant height with the maximum decrease in G2+S2 treatment in the varieties Gomati VU-89 (178.47 cm) and Pusa-578 (175.00 cm). The maximum GCV was recorded in S2 and G2 treatments in Gomati VU-89 (2.23%) and Pusa-578 (2.46%), respectively. In the variety Gomati VU-89, the highest h^2^ (91.42%) and GA (3.0%) were recorded in G1+S1 and S2 treatment, respectively. In the variety Pusa-578, the highest h^2^ (92.57%) and GA (6.01%) were recorded in S1 and G2+S2 treatments, respectively (Tables 3, 4).

**Table 3:** Estimates of mean values ± standard error (*n* = 30), genotypic coefficient of variation (GCV %), heritability (h^2^%), and genetic advance as % of the mean (GA %) for 10 quantitative traits in M_3_ generation of cowpea variety Gomati VU-89.

**Characters**	**Treatment population**						
		**C**	**G1**	**G2**	**S1**	**S2**	**G1+S1**	**G2+S2**
Plant height	MEAN	184.75^a^ ± 0.18	181.73^cd^ ± 0.46	181.05^d^ ± 0.45	183.52^b^ ± 0.47	182.79^bc^ ± 0.45	179.80^e^ ± 0.29	178.47^f^ ± 0.35
	GCV%	0.43	1.62	1.58	1.59	2.23	1.64	1.40
	h^2^%	43.62	65.06	65.29	64.00	86.15	91.42	72.41
	GA	0.41	1.89	1.89	1.83	3.00	2.30	1.78
Days to flowering	MEAN	80.46^a^ ± 0.38	79.06^bc^ ± 0.29	77.90^d^ ± 0.28	78.96^bc^ ± 0.25	78.73^c^ ± 0.25	79.73^ab^ ± 0.30	77.46^d^ ± 0.23
	GCV%	1.66	3.02	2.57	2.31	2.47	3.16	2.31
	h^2^%	30.81	79.73	71.29	74.04	76.56	81.46	78.92
	GA	2.43	7.12	5.73	5.24	5.72	7.54	5.43
Days to maturity	MEAN	151.40^a^ ± 0.49	148.83^b^ ± 0.57	147.56^bc^ ± 0.60	147.80^bc^ ± 0.60	147.03^c^ ± 0.55	147.93^bc^ ± 0.53	146.53^c^ ± 0.36
	GCV%	0.92	2.30	2.79	3.01	2.64	2.74	2.21
	h^2^%	22.61	62.02	69.71	74.81	72.23	76.68	86.38
	GA	1.16	4.78	6.15	6.87	5.93	6.35	5.44
Pods per plant	MEAN	60.86^d^ ± 0.42	68.10^ab^ ± 1.04	66.50^bc^ ± 1.04	63.80^c^ ± 1.10	66.10^bc^ ± 0.95	67.06^b^ ± 1.10	70.16^a^ ± 1.00
	GCV%	3.66	14.79	15.08	16.25	13.47	16.40	13.30
	h^2^%	54.74	89.95	89.66	88.52	88.19	91.66	88.08
	GA	7.16	37.03	37.70	40.36	33.41	41.45	32.95
Branches per plant	MEAN	8.86^e^ ± 0.0.14	11.63^b^ ± 0.26	11.43^bc^ ± 0.24	12.53^a^ ± 0.32	11.53^bc^ ± 0.24	11.20^d^ ± 0.25	11.70^b^ ± 0.25
	GCV%	7.23	18.41	18.08	19.15	16.61	19.12	18.37
	h^2^%	45.12	81.41	83.30	82.44	77.80	82.44	82.91
	GA	12.82	43.86	43.57	45.83	38.69	45.85	44.16
Seeds per pod	MEAN	11.80^bcd^ ± 0.08	12.60^abc^ ± 0.14	12.43^abc^ ± 0.16	13.00^a^ ± 0.19	12.70^ab^ ± 0.24	12.26^bcd^ ± 0.23	12.06^cd^ ± 0.20
	GCV%	4.64	10.98	12.00	13.48	18.06	19.03	16.17
	h^2^%	64.28	90.45	86.98	87.16	88.40	90.51	88.40
	GA	9.82	27.64	29.55	33.22	44.84	47.81	40.15
Seed weight	MEAN	12.90^c^ ± 0.04	13.91^a^ ± 0.15	13.30^bc^ ± 0.17	13.90^a^ ± 0.20	13.45^ab^ ± 0.18	13.40^ab^ ± 0.15	13.03^bc^ ± 0.18
	GCV%	1.96	9.08	11.93	12.24	12.93	13.52	12.11
	h^2^%	60.73	82.28	85.30	80.76	88.80	90.64	90.60
	GA	4.05	21.76	29.09	29.05	32.16	33.99	30.82
Pod length	MEAN	29.08^f^ ± 0.16	31.70^e^ ± 0.13	32.32^cd^ ± 0.18	32.17^de^ ± 0.21	32.85^bc^ ± 0.21	33.28^ab^ ± 0.26	33.74^a^ ± 0.25
	GCV%	1.08	3.32	4.17	5.44	4.83	6.27	5.63
	h^2^%	11.20	79.72	75.73	81.76	76.33	78.77	75.94
	GA	0.96	7.83	9.59	13.00	11.14	14.70	12.95
Plant yield	MEAN	92.64^f^ ± 0.42	119.27^a^ ± 1.02	110.52^b^ ± 0.64	115.28^b^ ± 0.84	112.90^c^ ± 0.62	106.72^e^ ± 0.65	109.99^d^ ± 0.68
	GCV%	2.47	9.41	6.06	7.69	6.05	6.39	6.52
	h^2^%	56.32	96.01	93.27	94.06	95.95	93.98	93.69
	GA	4.91	24.34	15.45	19.70	15.64	16.35	16.67
Harvest index	MEAN	28.51^f^ ± 0.31	42.63^a^ ± 0.56	40.32^bc^ ± 0.54	41.61^ab^ ± 0.52	39.27^cd^ ± 0.46	38.43^d^ ± 0.45	36.45^e^ ± 0.39
	GCV%	2.88	13.04	11.43	12.10	11.68	11.22	9.58
	h^2^%	20.26	91.23	83.17	89.45	90.99	88.84	85.46
	GA	3.43	32.88	27.52	30.22	29.42	27.92	23.39

*Means followed by the same letter is not different at a 5% level of significance, based on the DMRT*.

**Table 4:** Estimates of mean values ± standard error (*n* = 30), genotypic coefficient of variation (GCV %), heritability (h^2^%), and genetic advance as % of the mean (GA %) for 10 quantitative traits in M_3_ generation of cowpea variety Pusa-578.

**Characters**	**Treatment population**							
		**C**	**G1**	**G2**	**S1**	**S2**	**G1+S1**	**G2+S2**
Plant height	MEAN	181.91^a^ ± 0.24	178.00^c^ ± 0.34	176.50^d^ ± 0.50	180.01^b^ ± 0.30	178.41^c^ ± 0.38	177.60^c^ ± 0.39	175.00^e^ ± 0.45
	GCV%	0.66	1.80	2.46	1.73	2.13	1.97	2.42
	h^2^%	49.51	87.64	83.35	92.57	91.36	85.23	88.11
	GA	1.22	4.46	5.93	4.41	5.38	4.81	6.01
Days to flowering	MEAN	88.00^a^ ± 0.44	86.00^bcd^ ± 0.52	85.80^cd^ ± 042	85.50^d^ ± 0.50	87.20^abc^ ± 0.39	87.30^ab^ ± 0.54	86.50^bcd^ ± 0.42
	GCV%	2.07	5.15	4.56	5.42	4.27	5.78	4.00
	h^2^%	40.00	83.09	87.05	87.92	89.01	88.28	81.56
	GA	3.46	12.40	11.25	13.42	10.64	14.34	9.55
Days to maturity	MEAN	160.53^a^ ± 0.53	153.80^c^ ± 0.46	154.23^bc^ ± 0.51	155.50^bc^ ± 0.57	155.10^bc^ ± 0.58	154.53^bc^ ± 0.64	155.93^b^ ± 0.60
	GCV%	0.97	2.55	2.80	2.93	3.10	2.76	3.28
	h^2^%	24.22	83.41	83.00	79.44	81.69	68.42	82.74
	GA	1.26	6.16	6.74	6.91	7.40	6.04	7.89
Pods per plant	MEAN	41.23^e^ ± 0.29	47.06^bc^ ± 0.77	51.10^a^ ± 0.81	49.03^ab^ ± 0.93	45.95^cd^ ± 0.74	48.03^bc^ ± 0.87	43.93^d^ ± 0.89
	GCV%	3.52	14.46	12.91	16.99	13.79	16.01	15.68
	h^2^%	51.02	84.69	80.13	85.73	82.94	85.22	77.86
	GA	6.64	35.17	30.51	41.53	33.15	39.03	36.53
Branches per plant	MEAN	11.93^f^ ± 0.22	14.50^de^ ± 0.29	18.06^a^ ± 0.33	15.53^bc^ ± 0.41	13.83^e^ ± 0.34	16.06^b^ ± 0.34	14.90^cd^ ± 0.31
	GCV%	9.24	20.00	17.48	24.37	22.25	18.24	17.04
	h^2^%	48.60	90.99	88.21	87.50	85.23	82.61	79.46
	GA	17.00	50.38	43.35	60.20	54.24	43.78	40.11
Seeds per pod	MEAN	9.50^bc^ ± 0.10	10.80^ab^ ± 0.17	10.30^b^ ± 0.19	11.10^a^ ± 0.18	10.20^b^ ± 016	10.30^b^ ± 0.19	9.90^bc^ ± 016
	GCV%	5.50	14.47	17.73	12.88	12.15	15.43	12.93
	h^2^%	51.11	85.78	87.50	78.89	77.27	81.55	79.28
	GA	10.39	35.38	43.79	30.20	28.21	36.78	30.41
Seed weight	MEAN	20.50^c^ ± 0.22	22.20^a^ ± 0.40	20.60^c^ ± 0.32	21.50^ab^ ± 0.31	21.60^ab^ ± 0.30	21.40^ab^ ± 0.29	21.80^bc^ ± 0.36
	GCV%	6.30	14.93	13.45	12.32	11.73	12.61	13.07
	h^2^%	60.45	81.84	82.79	82.70	82.75	85.96	78.09
	GA	12.93	35.67	32.31	29.59	28.18	30.88	30.50
Pod length	MEAN	23.07^f^ ± 0.14	25.49^e^ ± 0.21	29.59^a^ ± 0.25	28.71^b^ ± 0.30	26.40^d^ ± 0.24	28.18^bc^ ± 0.31	27.98^c^ ± 0.24
	GCV%	3.00	7.37	6.91	8.72	7.67	9.93	7.72
	h^2^%	49.23	83.53	80.41	82.02	81.09	85.99	84.96
	GA	5.56	17.79	16.36	20.86	18.24	24.32	18.79
Plant yield	MEAN	80.29^g^ ± 0.53	112.83^b^ ± 0.77	108.42^a^ ± 0.64	117.01^a^ ± 0.98	101.25^e^ ± 0.67	105.90^d^ ± 0.97	94.80^f^ ± 0.57
	GCV%	3.01	7.02	5.94	8.22	6.23	9.36	6.32
	h^2^%	45.05	92.60	91.93	90.47	88.13	92.76	93.55
	GA	5.34	17.85	15.04	20.66	15.45	23.82	16.14
Harvest index	MEAN	29.86^d^ ± 0.22	40.54^a^ ± 0.58	35.87^c^ ± 0.51	38.43^b^ ± 0.47	35.76^c^ ± 0.41	36.39^c^ ± 0.39	37.07^c^ ± 0.47
	GCV%	2.76	13.55	8.45	11.71	8.99	9.41	9.64
	h^2^%	34.26	88.74	60.77	88.63	77.69	83.94	75.76
	GA	4.27	33.71	17.40	29.11	20.92	22.76	22.16

*Means followed by the same letter is not different at a 5% level of significance, based on the DMRT*.

#### Days to Flowering

In M_2_ generation, the mean number of days to flowering decreased significantly by about 3 days in G2 and G1 treatment in the varieties Gomati VU-89 and Pusa-578, respectively. The highest GCV was recorded in G2+S2 and G1+S1 treatments in Gomati VU-89 (3.95%) and Pusa-578 (6.21%), respectively. The highest h^2^ was recorded in G1+S1 and G1 treatments in the varieties Gomati VU-89 (78.57%) and Pusa-578 (82.08%), respectively. The maximum GA was recorded in G2+S2 and G1+S1 treatments in Gomati VU-89 (9.26%) and Pusa-578 (14.80%), respectively.

In the M_3_ generation, the flowering was earlier by 3.0 days in G2+S2 treatment and 2.5 days in S1 treatment in Gomati VU-89 and Pusa-578, respectively. The maximum GCV was recorded in G1+S1 treatment in the varieties Gomati VU-89 (3.16%) and Pusa-578 (5.78%), respectively. The highest h^2^ was recorded in G1+S1 and S2 treatments in Gomati VU-89 (81.46%) and Pusa-578 (89.01%), respectively. The maximum GA was recorded in G1+S1 treatment in Gomati VU-89 (7.54) and Pusa-578 (14.34) (Tables 3, 4).

#### Days to Maturity

In M_2_ generation, mutagen doses reduced the mean number of days to maturity with a maximum decrease by 11 and 10 days in G4+S4 treatment in the varieties Gomati VU-89 and Pusa-578, respectively. The highest GCV (4.12%; 4.77%) and GA (10.10%; 11.25%) were recorded in G1+S1 and S1 treatment in Gomati VU-89 and Pusa-578, respectively. The highest h^2^ was recorded in G2+S2 treatment in the varieties Gomati VU-89 (86.84%) and Pusa-578 (79.62%), respectively (Tables 1, 2).

Data recorded on mean values revealed a significant gain in decreasing the maturity period by 4.86 days with G2+S2 treatment in the variety Gomati VU-89 and 6.73 days with G1 treatment in the variety Pusa-578 in M_3_ generation. The highest GCV (3.01%) and GA (6.87%) were recorded in the S1 treatment, and the highest h^2^ (86.38%) was recorded in the G2+S2 treatment in the variety Gomati VU-89. The highest GCV (3.28%) and GA (7.89%) were recorded in the G2+S2 treatment, and the highest h^2^ (83.41%) was recorded in the G1 treatment in the variety Pusa-578 (Tables 3, 4).

#### Pods per Plant

In M_2_ generation, the highest increase in the mean number of pods per plant was recorded in G2+S2 and G2 treatment in the varieties Gomati VU-89 (68.03) and Pusa-578 (48.56), respectively. The highest GCV was recorded in G1 and G2 treatment in Gomati VU-89 (13.64%) and Pusa-578 (10.60%), respectively. In the variety Gomati VU-89, the maximum h^2^ (90.39%) and GA (34.24%) were recorded in G1 treatment, while in the variety Pusa-578, the highest h^2^ (81.38%), and GA (25.26%) were recorded in the G2 treatment (Tables 1, 2).

In M_3_ generation, the maximum increase in the mean number of pods per plant was recorded in G2+S2 and G2 treatment in the varieties Gomati VU-89 (70.16) and Pusa-578 (51.10), respectively. Maximum GCV (16.40%), h^2^ (91.66%), and GA (41.45%) were recorded in G1+S1 treatment in the variety Gomati VU-89. In the variety Pusa-578, maximum GCV (16.99%), h^2^ (85.73%), and GA (41.53%) were recorded in S1 treatment (Tables 3, 4).

#### Branches per Plant

In M_2_ generation, the maximum increase in the mean number of branches per plant was recorded in G2+S2 and G2 treatments in the varieties Gomati VU-89 (12.46) and Pusa-578 (15.03), respectively. The highest GCV was noted in G2+S2 treatment in Gomati VU-89 (18.16%) and Pusa-578 (16.40%). The highest h^2^ (79.06%; 85.24%) and GA (42.62%; 38.27%) were recorded in G2+S2 and G1+S1 treatment in the varieties Gomati VU-89 and Pusa-578, respectively (Tables 1, 2).

In M_3_ generation, the maximum increase in branches per plant was recorded in S1 and G2 treatment in the varieties Gomati VU-89 (12.53) and Pusa-578 (18.06), respectively. Maximum GCV was recorded in S1 treatment in Gomati VU-89 (19.15%) and Pusa-578 (24.37%). The highest GA was recorded in G1+S1 and S1 treatment in the varieties Gomati VU-89 (45.85%) and Pusa-578 (60.20%), respectively. The maximum h^2^ was recorded in G2 and G1 treatment in Gomati VU-89 (83.30%) and Pusa-578 (90.99%), respectively (Tables 3, 4).

#### Seeds per Pod

In M_2_ generation, the highest increase in the mean number of seeds per pod was recorded in S1 treatment in the varieties Gomati VU-89 (12.80) and Pusa-578 (11.00). The highest GCV was recorded in G1+S1 and S1 treatment in Gomati VU-89 (17.46%) and Pusa-578 (12.70%), respectively. The highest h^2^ (80.21%; 80.39%) and GA (41.28%; 30.06%) were recorded in G1+S1 and S1 treatment in the varieties Gomati VU-89 and Pusa-578, respectively (Tables 1, 2).

In M_3_ generation, the maximum increase in the mean number of seeds per pod was recorded in S1 treatment in the varieties Gomati VU-89 (13.00) and Pusa-578 (11.10). The highest GCV (19.03%), h^2^ (90.51%), and GA (47.81%) were recorded in G1+S1 treatment in the variety Gomati VU-89, whereas in the variety Pusa-578, the highest GCV (17.73%), h^2^ (87.50%) and GA (43.79%) were recorded in G2 treatment (Tables 3, 4).

#### Seed Weight (g)

In M_2_ generation, the maximum mean weight of 100 random seeds was recorded in S1 treatment in the varieties Gomati VU-89 (13.70 g) and Pusa-578 (22.40 g). In the variety Gomati VU-89, the highest GCV (14.51%), h^2^ (79.75%), and GA (34.21%) were recorded in G2+S2 treatment, whereas in the variety Pusa-578, the highest GCV (13.43%), h^2^ (82.22%), and GA (32.17%) were recorded in G2 treatment (Tables 1, 2).

In M_3_ generation, the maximum increase in mean 100 random seed weight was recorded in G1 treatment in the varieties Gomati VU-89 (13.91 g) and Pusa-578 (22.20 g). The highest GCV (13.52%), h^2^ (90.64%), and GA (33.99%) were recorded in G1+S1 treatment in the variety Gomati VU-89. In the variety Pusa-578, the highest GCV (14.93%) and GA (35.67%) were recorded in G1 treatment, and the highest h^2^ (85.96%) was obtained in G1+S1 treatment (Tables 3, 4).

#### Pod Length (cm)

The maximum increase in mean pod length was recorded in G2+S2 and G2 in the varieties Gomati VU-89 (32.03 cm) and Pusa-578 (27.72 cm), respectively. The maximum GCV was recorded in G1+S1 and G2 treatment in the varieties Gomati VU-89 (4.71%) and Pusa-578 (8.41%), respectively. The highest h^2^ (81.27%; 79.80%) and GA (32.42%; 19.85%) were recorded in S1 and G2 treatments in the varieties Gomati VU-89 and Pusa-578, respectively (Tables 1, 2).

In M_3_ generation, the maximum increase in mean pod length was recorded in G2+S2 and G2 treatment in the varieties Gomati VU-89 (33.74) and Pusa-578 (29.59), respectively. The highest GCV (6.27%; 9.93%) and GA (14.70%; 24.32%) were recorded in G1+S1 treatment in the varieties Gomati VU-89 and Pusa-578, respectively. The maximum h^2^ was recorded in S1 and G1+S1 treatment in Gomati VU-89 (81.76%) and Pusa-578 (85.99%), respectively (Tables 3, 4).

#### Plant Yield (g)

In M_2_ generation, the highest increase in mean plant yield was recorded in G1 and S1 treatment in the varieties Gomati VU-89 (112.00 g) and Pusa-578 (110.63 g), respectively. The maximum GCV (7.43%; 6.35%), h^2^ (82.46%; 91.52%) and GA (17.81%; 16.50%) were recorded in G1 and G1+S1 treatment in the varieties Gomati VU-89 and Pusa-578, respectively (Tables 1, 2).

In M_3_ generation, the highest mean plant yield was noted in G1 and S1 treatments in the varieties Gomati VU-89 (119.27 g) and Pusa-578 (117.01 g), respectively. In the variety Gomati VU-89, maximum GCV (9.41%), h^2^ (96.01%), and GA (24.34%) were recorded in the G1 treatment. On the contrary, as in the variety Pusa-578, maximum GCV (9.36%), and GA (23.82%) were recorded in the G1+S1 treatment, and the highest h^2^ (93.55%) was recorded in the G2+S2 treatment (Tables 3, 4).

#### Harvest Index per Plant (%)

In M_2_ generation, the maximum increase in mean harvest index per plant was recorded in G1 treatment in the varieties Gomati VU-89 (41.25%) and Pusa-578 (38.51%). The highest GCV (18.17%) was recorded in the G2 treatment, while maximum h^2^ (83.23%) and GA (23.93%) were recorded in the S1 treatment in the variety Gomati VU-89. In the variety Pusa-578, the highest GCV (7.73%), h^2^ (71.90%), and GA (17.32%) were recorded in S1 treatment (Tables 1, 2).

In M_3_ generation, the highest harvest index was noted in G1 treatment in the varieties Gomati VU-89 (42.63%) and Pusa-578 (40.54%). The maximum GCV (13.04%; 13.55%), h^2^ (91.23%; 88.74%), and GA (32.88%; 33.71%) were recorded in G1 treatment in the varieties Gomati VU-89 and Pusa-578, respectively (Tables 3, 4).

### Correlation Analysis: Relationship Among Yield and Yield Attributing Traits

**Figure 6:**
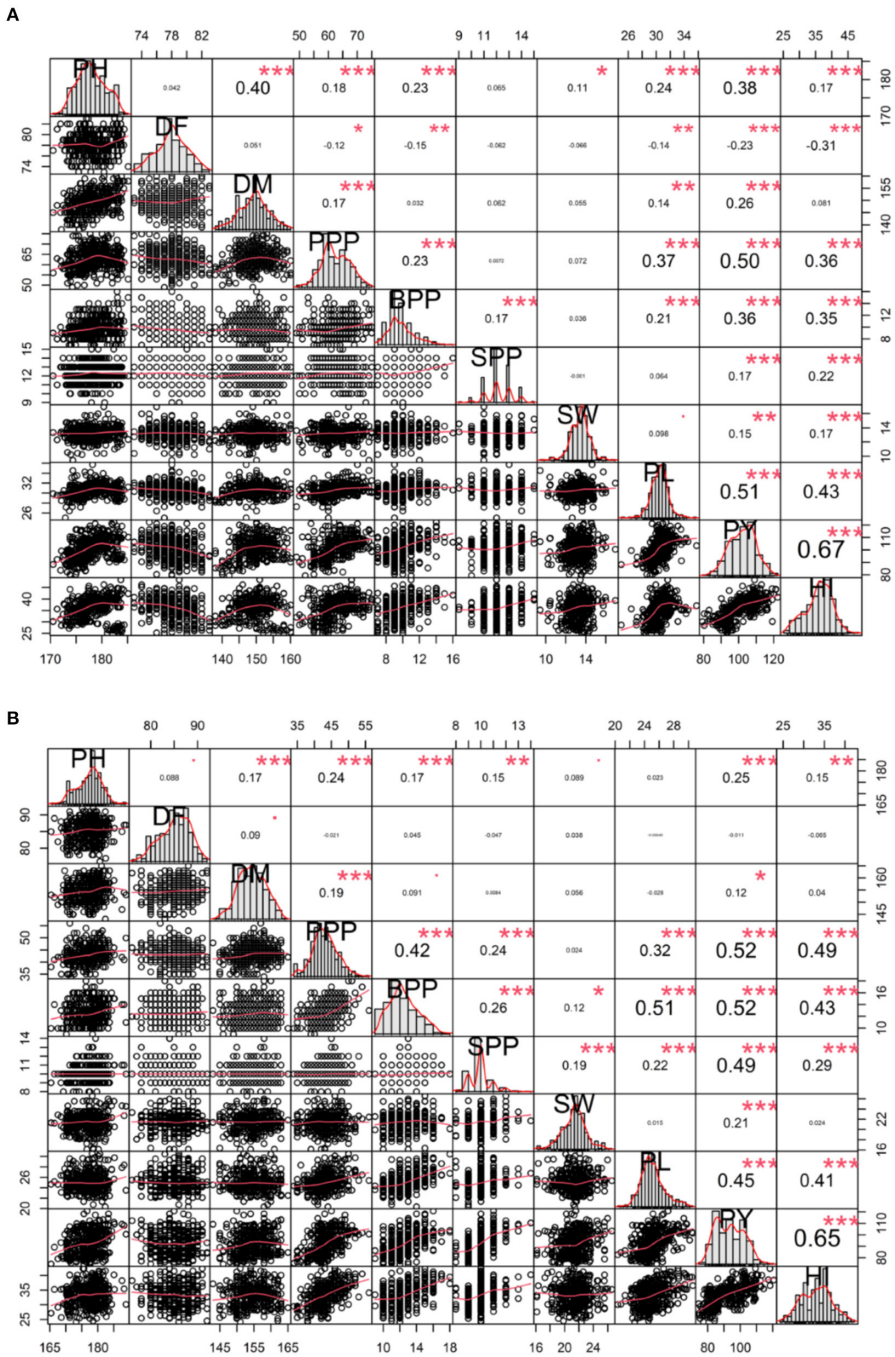
Correlation analysis between yield and yield attributing traits **(A)** variety Gomati VU-89 and **(B)** variety Pusa-578. PY, Plant yield; PPP, Pods per plant; BPP, Branches per plant; SPP, Seeds per pod; SW, Seed weight; PL, Pod length; HI, Harvest index. Significant differences are indicated as ***P < 0.001, **P < 0.01, and *P < 0.05.

In-plant breeding programs, assessing the relationship between yield and yield attributing traits is imperative. Spearman's correlation analysis helped us to visualize the relationship between and among quantitative traits. In the variety Gomati VU-89, the plant yield showed a significant positive phenotypic correlation with harvest index followed by pod length, pods per plant, plant height, branches per pod, days to maturity, seeds per pod, seed weight, and a significant negative correlation with days to flowering (Figure 6). In the variety Pusa-578, the plant yield showed substantial positive phenotypic correlation with harvest index followed by pods per plant, branches per plant, seeds per pod, pod length, plant height, seed weight, days to maturity, and non-significant negative correlation with days to flowering (Figure 6).

#### Correlation Analysis: Concurrent Increase in Yield and Nutrient Density

The results revealed a positive correlation between plant yield and physiological parameters such as NRA, chlorophyll, carotenoids, protein, iron, zinc, and copper in M_4_ high yielding mutant lines. Plant yield correlated positively with all the parameters in Gomati VU-89-C, Gomati VU-89-D, Gomati VU-89-F, and Gomati VU-89-G, except (i) NR activity in Gomati VU-89-A and Gomati VU-89-E, and (ii) copper contents in Gomati VU-89-B. Plant yield showed a positive correlation with all the parameters in Pusa-578-C except (i) zinc and copper contents in Pusa-578-A, (ii) iron and zinc contents in Pusa-578-B, and (iii) iron contents in Pusa-578-D (Supplementary Table 7).

### Path Analysis: Direct and Indirect Impact of Yield Attributing Traits on Yield

**Figure 7:**
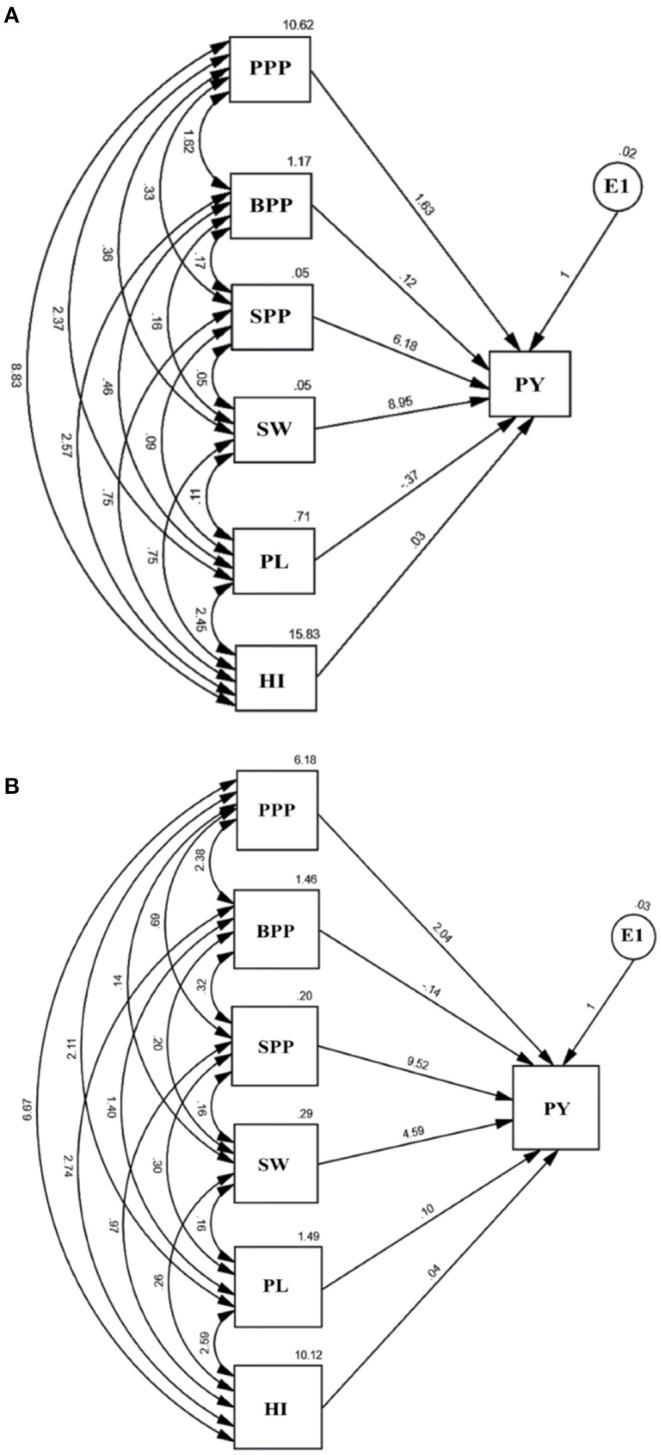
Path analysis in **(A)** variety Gomati VU-89 and **(B)** variety Pusa-578 showing the interrelationship between PY, plant yield, and yield attributes, viz. PPP, Pods per plant; BPP, Branches per plant; SPP, Seeds per pod; SW, Seed weight; PL, Pod length; HI, Harvest index and E1: Residual.

The path analysis allowed us to visualize the direct and indirect impact of yield attributing traits (Figure 7). The causal variables included pods per plant, branches per plant, seeds per pod, seed weight, pod length, and harvest index, while plant yield was treated as a resultant variable and E1 as a residual factor, unaccounted and independent of the other variables. The results revealed that seed weight and seeds per pod possess a maximum positive direct effect on plant yield while pod length and branches per plant negatively impacted yield. Path analysis indicated that contributions of trait toward yield followed an increasing trend, i.e., pod length<harvest index<branches per plant<pods per plant<seeds per pod<seed weight in the variety Gomati VU-89 and branches per plant<harvest index<pod length<pods per plant<seed weight<seeds per pod in the variety Pusa-578.

#### Plant Yield vs. Pods per Plant

The direct effect of pods per plant on plant yield was 1.63 in the variety Gomati VU-89 and 2.04 in the variety Pusa-578. Pods per plant showed a positive indirect impact on plant yield through branches per plant (1.62; 2.38), seeds per pod (0.33; 0.69), seed weight (0.36; 0.14), pod length (2.37; 2.11), and harvest index (8.83; 6.67) in the varieties Gomati VU-89 and Pusa-578, respectively.

#### Plant Yield vs. Branches per Plant

The direct impact of branches on plant yield was 0.12 in the variety Gomati VU-89 and−0.14 in the variety Pusa-578. It showed a positive indirect impact on plant yield through seeds per pod (0.17; 0.32), seed weight (0.16; 0.20), pod length (0.46; 1.40), and harvest index (2.57; 2.47) in the varieties Gomati VU-89 and Pusa-578, respectively.

#### Plant Yield vs. Seeds per Pod

The direct impact of seeds per pod on plant yield was 6.18 in the variety Gomati VU-89 and 9.52 in the variety Pusa-578. It had positive indirect impact on plant yield through branches per plant (0.17; 0.32), seed weight (0.05; 0.16), pod length (0.09; 0.30), harvest index (0.75; 0.97) in the varieties Gomati VU-89 and Pusa-578, respectively.

#### Plant Yield vs. Seed Weight

The direct impact of seed weight on plant yield was 8.95 in the variety Gomati VU-89 and 4.59 in the variety Pusa-578. Seed weight showed a positive indirect impact on plant yield through pod length (0.11; 0.16) and harvest index (0.75; 0.26) in the varieties Gomati VU-89 Pusa-578, respectively.

#### Plant Yield vs. Pod Length

The direct impact of pod length on plant yield was −0.37 in the variety Gomati VU-89) and 0.10 in the variety Pusa-578. Pod length showed a strong positive indirect impact on plant yield through harvest index (2.45; 2.59) in the varieties Gomati VU-89 and Pusa-578, respectively.

#### Plant Yield vs. Harvest Index

The direct impact of harvest index on plant yield was 0.03 in the variety Gomati VU-89) and 0.04 in the variety Pusa-578.

### Multivariate Analysis

#### Hierarchical Cluster Analysis Delineated Control and Treated Cowpea

**Figure 8:**
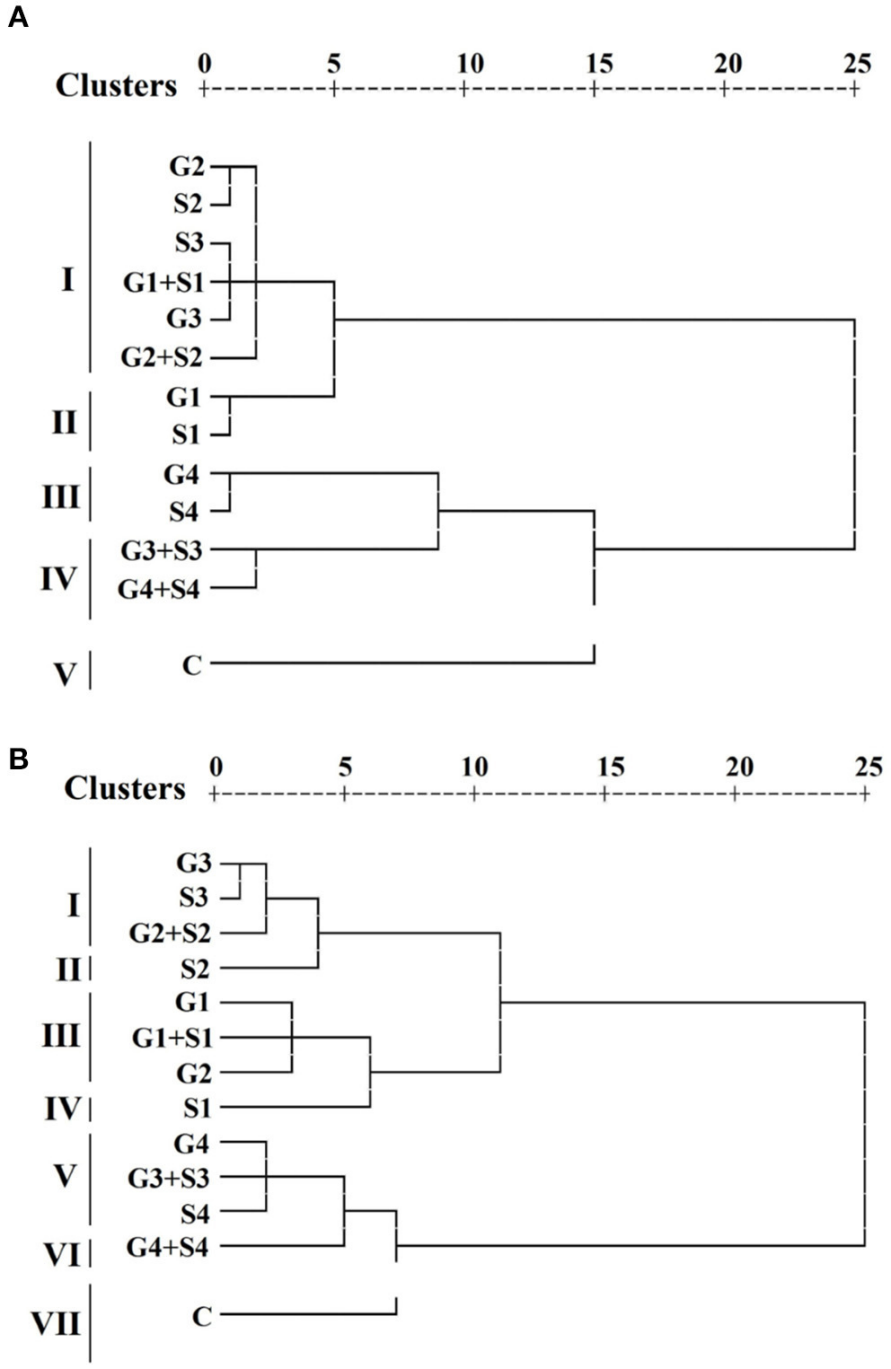
Dendrogram clustering based on quantitative traits in **(A)** variety Gomati VU-89 and **(B)** variety Pusa-578.

Hierarchical cluster analysis grouped treated and untreated populations into different clusters. A dendrogram was constructed based on the average linkage between the populations for each variety (Figure 8). HCA revealed that treated and control populations were grouped into five clusters I, II, III, IV, and V. Cluster I comprises six treated populations. On the contrary, clusters II, III, and IV comprise two treated populations, and cluster V comprises one untreated population in the variety Gomati VU-89. HCA also divided the treated and untreated population into seven clusters I, II, III, IV, V, VI, and VII. Clusters I, III, and V comprise three treated populations, while clusters II, IV, and VI comprise one treated population, and cluster VII comprises one untreated population in the variety Pusa-578. Average values of 10 quantitative phenotypic traits for three groups among 13 populations per variety are furnished in Supplementary Table 8. Results revealed that cluster II had the maximum mean values for pods per plant (65.97; 44.9), branches per plant (11.35; 13.7), seeds per pod (12.47; 11.00), seed weight (13.49; 22.40), pod length (30.71; 26.72), plant yield (111.37; 110.64), and harvest index (40.84%; 37.54%) in the varieties Gomati VU-89 and Pusa-578, respectively. We recorded the maximum inter-cluster distance between clusters I and II in Gomati VU-89 (19.25) and Pusa-578 (25.45) (Supplementary Table 9), respectively.

The inter-population dissimilarity matrix showed a maximum Euclidean distance between G4+S4 and G1 treated population in the variety Gomati VU-89 (29.00), whereas in the variety Pusa-578, it was recorded between G4+S4 and S1 treated population (35.62), indicating that these populations are most dissimilar. The lowest Euclidean distance was recorded between G1+S1 and S3 treated population in the variety Gomati VU-89 (2.66) and S3 and G3 treated population (1.92) in the variety Pusa-578, indicating that these populations are most similar. The most distanced population were G1 (24.00) and S1 (29.39) in the varieties Gomati VU-89 and Pusa-578, respectively (Supplementary Table 10).

#### Principal Component Analysis: Simultaneous Analysis of Multiple Traits

**Figure 9:**
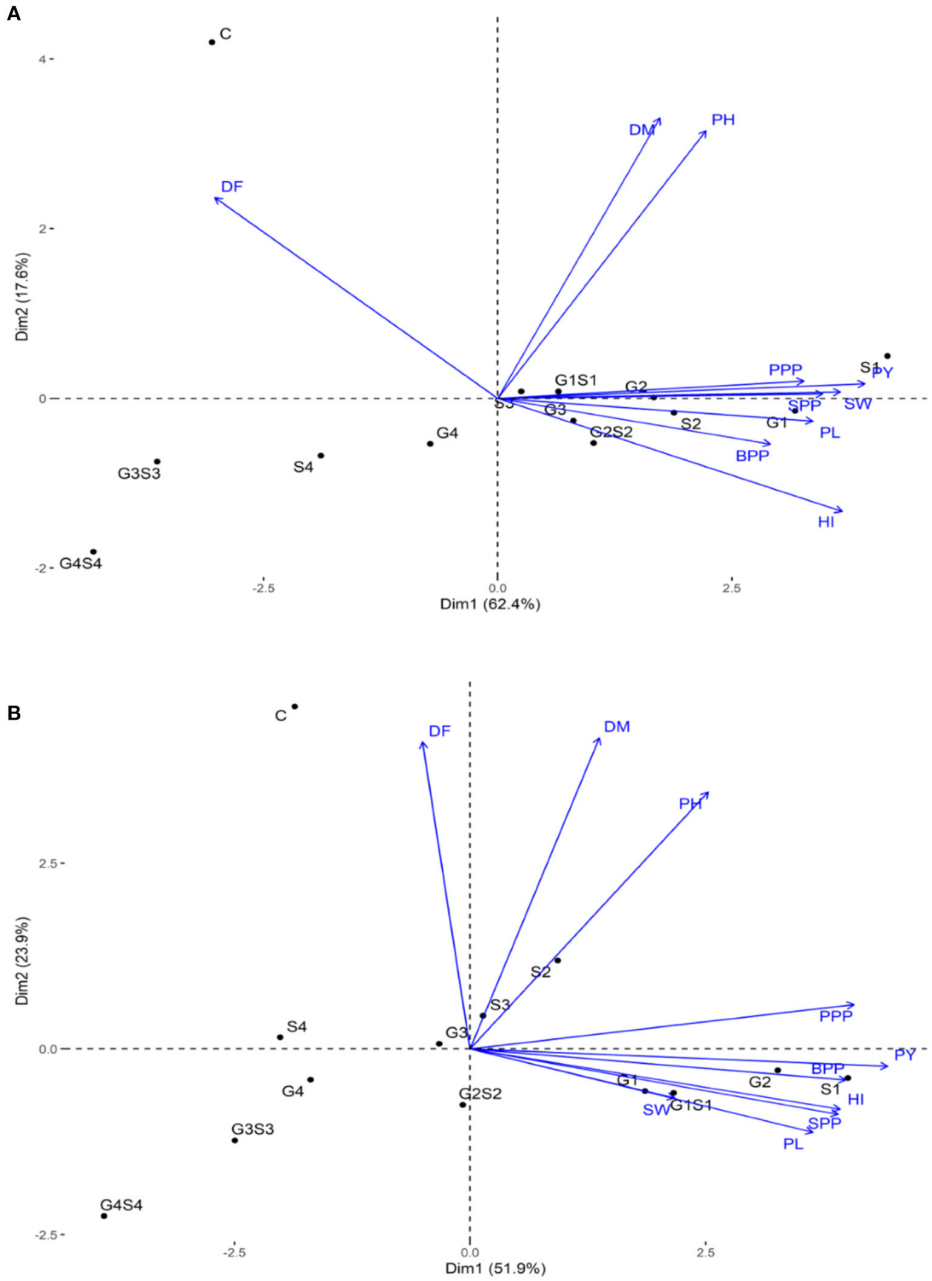
Principal component analysis in **(A)** variety Gomati VU-89 and **(B)** variety Pusa-578, showing effects of mutagens on various studied attributes of cowpea. DF, Days to flowering; DM, Days to maturity; PH, Plant height; PPP, Pods per plant; BPP, Branches per plant; SPP, Seeds per pod; SW, Seed weight; PL, Pod length; HI, Harvest index.

**Figure 10:**
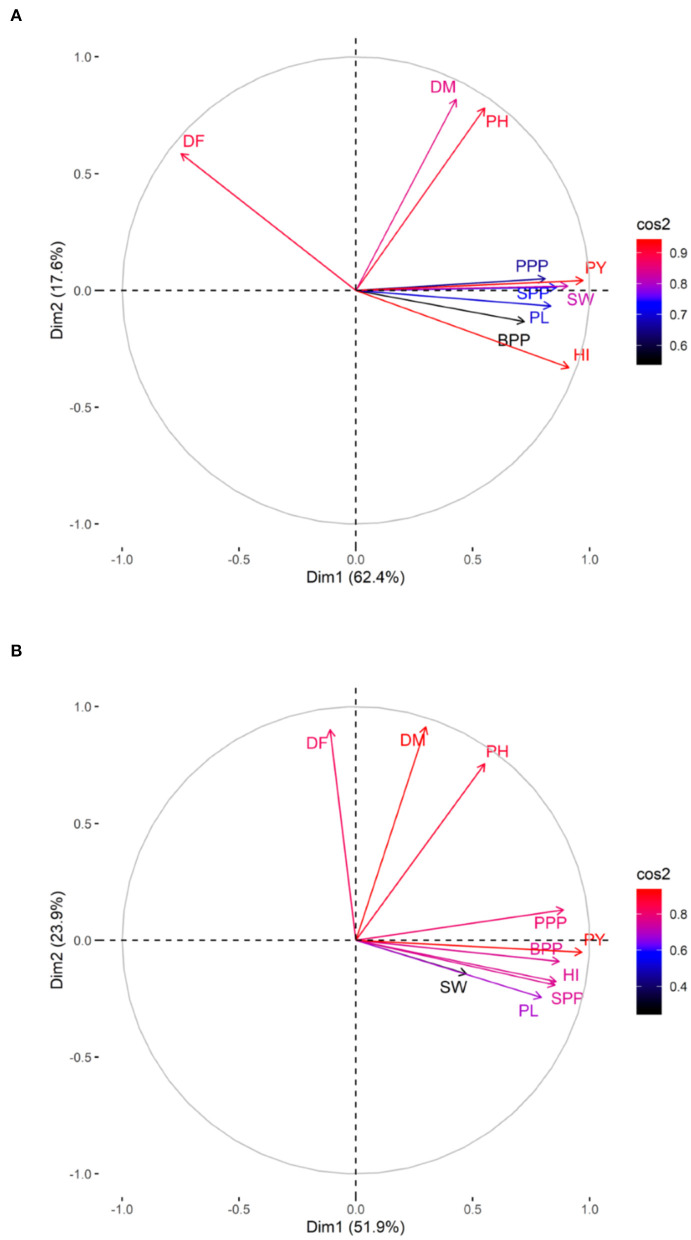
Quality of representation (cos2) of quantitative traits. **(A)** variety Gomati VU-89 and **(B)** variety Pusa-578, high cos2 values are colored in “red,” with mid cos2 values colored in “blue,” and low cos2 values with “black color.”

The PCA revealed lower and moderate mutagen treated populations viz., G1, G2; S1, S2, S3; G1S1, and G2S2 and higher mutagen treated populations viz., G4; S4; G3S3, and G4S4 lie on opposite quadrants in the biplot (Figure 9). In the variety Gomati VU-89, the PCA revealed that first two principal components explained 80% of the total variation (PC1 = 62.4%; PC2 = 17.6%), while as in the variety Pusa-578, the first two principal components explained 75.8% of the total variation (PC1 = 51.9%; PC2 = 23.9%). The magnitude of each quantitative trait in different treatments is shown in Figure 10, and the treatment on the same side of the quantitative trait has a higher value for that quantitative trait and vice versa. Cos2 indicates the quality of representation and contribution of quantitative traits (in %) to the principal components. Our results revealed that PY, HI, PH, DF, DM, SPP, and SW showed good representation, while BPP, PL, and PPP showed weak representation in the variety Gomati VU-89 (Figure 10). However, in the variety Pusa-578, all the traits except BPP showed good representation (Figure 10).

**Figure 11:**
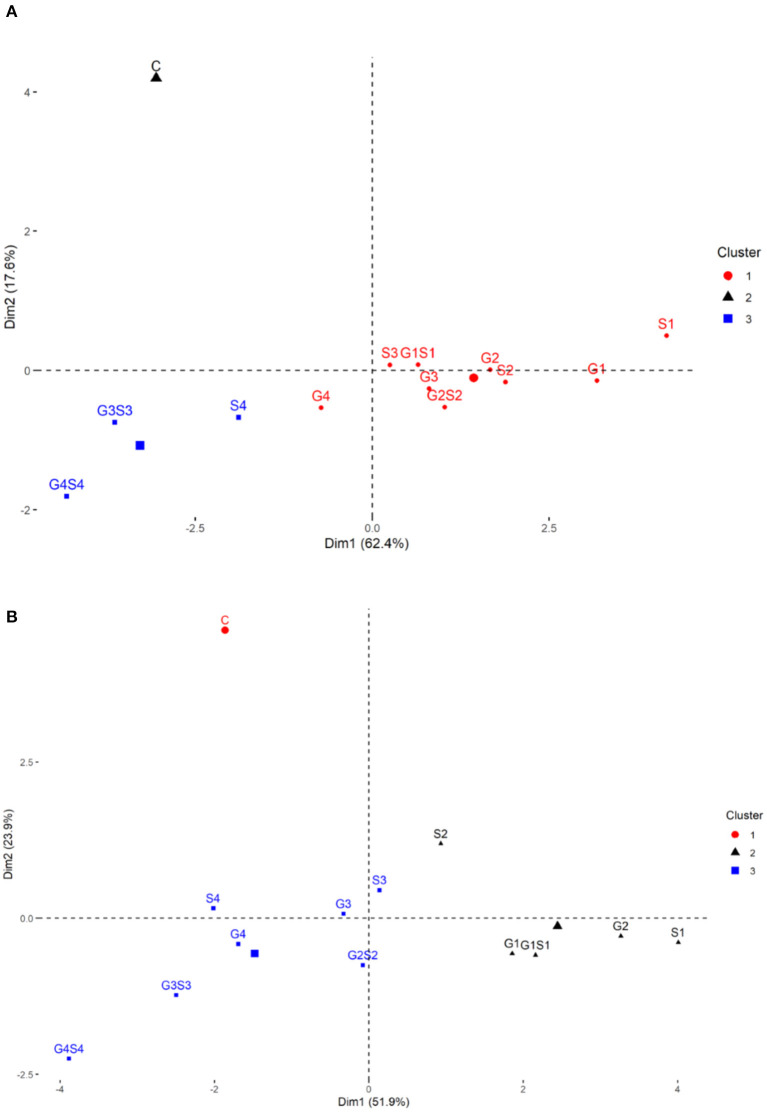
Clustering of mutagenized population based on the representation of quantitative traits: **(A)** variety Gomati VU-89 and **(B)** variety Pusa-578.

Based on the magnitude and representation of quantitative traits across different treatments, lower and moderate treated populations viz., G1, G2, G3, G4, S1, S2, S3, G1S1, and G2S2 formed the first cluster. In contrast, control and higher treated populations viz., S4, G3S3, and G4S4 formed second and third clusters, respectively, in the variety Gomati VU-89 (Figure 11). The control population forms the first cluster, and G1, G2, S1, S2, and G1S1 treated populations form the second cluster, while G3, G4, S3, S4, G2S2, G3S3, and G4S4 treated populations forms the third cluster in the variety Pusa-578 (Figure 11).

## Discussion

### Mutagenic Mode of Action

In the present study, we chose γ rays and SA due to their high effectiveness and efficiency in inducing different mutations. Among the physical mutagens, γ rays are mainly used to improve economically important yield and yield contributing traits (Celik and Atak, 2017). Gamma rays have successfully developed 1,716 mutants in different crop species (see text footnote 1). During irradiation, γ rays interact with the tissues and facilitate electron ionization and excitation that distorts the stable DNA structure. The distorted DNA structure eventually causes hydrolysis of chemical bonds linking nitrogenous bases with the backbone of DNA. Gamma rays also cause radiolysis of cellular water and lead to the formation of positively charged free radicals, viz. reactive oxygen species (ROS), ionized water (H_2_O^+^) hydroxyl radical (**·**OH), and other reactive nitrogen species (RNS). These free radicals attack the DNA constituents and induce changes in deoxyribose rings, DNA bases, DNA–DNA cross-links, and DNA–protein cross-links leading to the induction of alterations such as substitutions, deletions, and chromosomal aberrations (Reisz et al., 2014).

The synthesis of an organic metabolite, β-azidoalanine [N3–CH2–CH (–NH2)–COOH], an amino acid analog “L-azidoalanine” mediates the mutagenicity of SA. This organic metabolite interacts with DNA and induces AT → GC base pair transition and transversion (Khan et al., 2009) and chromosomal aberrations (Gruszka et al., 2012). Being capable of generating point mutation, SA alters many developmental, physiological, and metabolic activities of cowpea.

### Genetic Parameters and Their Response to Mutagen Doses

In mutation breeding, induction of desirable micro-mutations affecting the economically important quantitative traits like yield is a prime goal (Laskar et al., 2018b; Goyal et al., 2021a,b). In the present study, mutagen doses were successful in increasing the values of GCV, h^2^, and GA for all the quantitative traits. The increase in genetic parameters may be attributed to mutagens or pleiotropic effects of newly mutated genes. The results were in good agreement with the previous studies that have also reported enhanced GCV, h^2^, and GA in lentil mutant lines (Laskar and Khan, 2017). Our results revealed lower and moderate doses of γ rays and SA employed individually induced desirable mutations in quantitative traits. However, higher doses of combined γ rays and SA-induced undesirable mutations may be attributed to the synergistic effect of the mutagens. Therefore, it is recommended to expose the plant material individually to evade the harmful effects of combined mutagens. Different statistical measures have been employed in both M_2_ and M_3_ generations to assess variations in quantitative traits and genetic variability, considering the mutagen-induced genetic variability. The effects of mutagen doses on the quantitative traits and genetic parameters are discussed below:

### How Do Quantitative Traits Influence Total Cowpea Yield?

In the present study, decreased plant height in all mutagen-treated populations could be exploited in the future cowpea breeding program. The decreased plant height mutants are lodging resistant and can withstand the fast winds prevalent during the flowering season of cowpea. Anjana and Thimmaiah (2002) also reported dwarf mutants with a higher nitrogen-fixing ability in gamma irradiated cowpea. The mutagen-induced inhibition of mitotic divisions and expression of phytohormone synthesis genes may be ascribed to reducing plant height (Rao, 1988; Cheng et al., 2019). Besides plant height, mutagens reduced flowering time and depicted a scope for selecting early flowering mutants in the later generations. Dhakhanamoorthy et al. (2010) and Horn et al. (2016) also reported mutagen-induced earliness in cowpea varieties, and opinionated early flowering might be due to the physiological changes caused by mutagens. The flowering time and the entire maturity period were shorter in a few mutant lines observed in both generations. This short maturity period would help the cowpea escape the prevalent heatwaves during the pod filling stage. Adekola and Oluleye (2007) also reported early maturing mutants in gamma irradiated cowpea. Another desirable mutation was an increased branching capacity in mutagen treated population that might be due to rapid cell division, elongation, and synthesis of phytohormones or nucleic acids (Hanan et al., 2011). Essel et al. (2015); Khursheed et al. (2019) also reported the same effect of mutagens on the branching capacity.

Most importantly, we noticed that lower and medium mutagen doses increased the mean number of pods per plant, an important trait from the breeder's perspective. The previous studies in gamma-irradiated cowpea varieties also reported the same findings (Kumar et al., 2010). The physiological effects of lower and medium mutagen doses and their hydrolysis products may be attributed to the augmented number of pods. Interestingly a slight and non-significant increase was recorded in the mean number of seeds per pod in the M_2_ and M_3_ generations. This depicted apparent trait stability was also supported by earlier findings in mutagenized lentils (Laskar and Khan, 2017) and pigeon pea cultivars (Giri et al., 2010). In cowpea, seed weight is an important trait for predicting yield, therefore, we evaluated the effects of mutagens on the seed weight in both generations. Our results revealed that seed weight also increased in populations treated with lower and medium mutagen doses. The results were in line with the findings in mutagenized cowpea cultivars (Odeigah et al., 1998; Kumar et al., 2010). Besides seed weight, pod length is another essential trait that contributes to the seed yield as longer pod lengths accommodate more seeds (Horn et al., 2016). In the present study, almost all the mutagen doses induced a substantial increase in mean pod length that might be ascribed to the predominant prevalence of desirable mutations in the treated population. The same findings were also reported in earlier studies (Kashid and Kulthe, 2014). In the present study, a significant increase in plant yield was supported by previous reports on mutagenized cowpea varieties (Kumar et al., 2010; Horn et al., 2016). Since yield is a complex trait involving additive effects of several genes, it is challenging to identify the mutated genes that govern the increase in yield. To harness the full genetic potential of cowpea, evaluation of the harvest index is imperative (Adeyanju, 2009). The harvest index, a ratio of seed yield to dry weight, is used to evaluate the efficiency of allocation of assimilated photosynthate to the seeds (Sinclair, 1998). In the present study, increased harvest index in the lower and medium mutagen doses may be attributed to the mutagen-induced higher plant yield. The results were supported by the earlier works (Laskar and Khan, 2017).

### Interrelationship Among Yield and Yield Component Traits

In mutation breeding programs, correlation analysis between character pairs plays a vital role in determining the influence of yield attributes on total yield (Goyal et al., 2019). The direct selection for yield is rarely effective as it is a polygenic trait with a complex mode of inheritance. Small additive effects of multiple genes that govern the trait expression influence the correlations between quantitative traits. Therefore, it is imperative to emphasize on indirect selection, focusing on traits directly impacting yield. Achieving maximum selection gains require adequate knowledge of the correlation between yield and yield attributes. A trait showing a high correlation with another desired trait is considered better for indirect selection. In the present study, plant yields correlated positively with all the studied traits except days to flowering. The results were in good agreement with the previous studies (Silva et al., 2015; Laskar and Khan, 2017). The negative correlation between yield and flowering time is an important trait of interest in warm-season legumes like cowpea. Early flowering enables the cowpea to avoid heat stress-induced yield losses during the reproductive phase. Significant positive correlations between plant yield and pods per plant revealed that pods per plant contributed maximally toward plant yield, and plants with more pods tend to have a higher yield. The results were also supported by the earlier findings in chickpeas (Raina et al., 2017). Since the mode of selection for an untreated and treated population is the same, the observed differences in the correlation coefficients could be attributed to the effect of mutagens or altered pleiotropic effects of newly mutated genes.

### Is Biofortification Possible in High Yielding Cowpea Mutant Lines?

In the present study, a concurrent increase in yield and nutrient density was obtained in M_4_ high yielding mutant lines indicating a broad possibility of developing biofortified cowpea after subjecting to multi-location trails. Mutant lines with a high yield and micronutrient concentration provided an added benefit for future breeding programs. Besides the current scenario of food insecurity and widespread malnutrition, developing biofortified cowpea varieties could keep up with the nutritional demands of millions of undernourished people living in Asian and African countries. Gregoria (2002) also reported a concurrent improvement in nutrient density and yield in staple crops using conventional breeding strategies. Therefore, mutation breeding with the rich literature on optimum doses is a promising technique to generate biofortified mutants that would accomplish the nutritional targets and sustainable development goals that ensure zero hunger and a better future for all people worldwide.

### Path Analysis: Direct and Indirect Impact of Component Traits on Yield

The path analysis revealed that seed weight is one of the primary yield components due to its greatest direct and indirect impact on plant yield. The results agree with the previous findings that heavier seeds show a higher yield than lighter seeds (Elliott et al., 2008). Besides seed weight, seeds per pod also revealed a great direct impact on plant yield. Yang et al. (2016) also reported the same findings and suggested that crops with more seeds per pod are expected to yield more than crops with fewer seeds per pod. It is important to mention that characteristics like plant height, days to flowering, and days to maturity revealed a low and negligible effect on the plant yield and hence were not included in the path analysis.

In the present study, correlation and path analysis revealed that seed weight and seeds per pod are the main yield components. However, contradicting conclusions on various traits were also recorded. For instance, the correlation between plant yield and pod length was highly significant and positive, indicating that pod length greatly influenced plant yield. In contrast, path analysis revealed pod length as an unimportant factor influencing plant yield. The noticeable disagreement between correlation and path analysis may be attributed to the fact that the correlation calculates mutual association only without considering the causes; however, path analysis specifies the causes and calculates their relative significance. Hence to get an accurate association between traits, it is imperative to study correlation and path analysis.

### Multivariate Analysis: Reducing Complex Data to Simple in an Error-Free Process

Analyzing numerous phenotypic characters is a hectic and error-prone process that may influence selection output; therefore, multivariate analysis is vital for proper selection (Muduli and Misra, 2008). It helps a breeder to reduce complex data and enhances breeding precision. In multivariate analysis, HCA and PCA are the primary tools for aiding the correct selection (Malek et al., 2014). In the present study, HCA divided mutagenized and control populations into separate clusters, indicating that mutagenic treatments induced heterogeneous populations in the background of two parental varieties. Genetically diverse populations were grouped within the different clusters. Therefore, mutants selected from different clusters could be advanced to subsequent generations in cowpea improvement programs aimed at increasing genetic diversity/variability.

The inter-cluster distance helped us to visualize the spectrum of variability. The lesser distance between the clusters indicates a narrow spectrum of variability in the segregating generations and vice versa. In the present study, maximum mean values for most quantitative traits were recorded in cluster II. Hence, the population forming cluster II could be evaluated for selecting high yielding mutant lines. The results were in good agreement with the earlier findings of Laskar and Khan (2017) that reported maximum values of quantitative traits associated with one of the clusters in lentil mutant lines. HCA also revealed that G2 and G3 populations were most mutated and distinct with respect to the control population. Therefore, these populations could be advanced to subsequent generations for a further selection of high-yielding mutant lines.

PCA is considered helpful for selecting high-yielding mutant lines for breeding programs (Afuape et al., 2011). A significant positive correlation was observed between plant yield and pods per plant, seeds per pod, and branches per plant. However, days to flowering correlated negatively with yield and yield attributing traits, suggesting that earliness is a desirable trait in breeding programs. The PCA also equips breeders in identifying the range of variability contributed by different yield attributes. In the present study, plant yield contributed maximally toward genetic variability, and hence it might be prioritized in selecting high yielding mutant lines. The PCA divided the mutagenized population into groups scattered in all quadrants, indicating a wide genetic variability induced in phenotypic traits. The mutagenized population treated with lower and moderate mutagen doses remained around the origin of PC2, indicating genetically similar mutants. However, the mutagenized population treated with higher mutagen doses remained at extreme positions from the origin in the PCA biplot, indicating genetically divergent mutants. Therefore, lower and higher mutagen doses effectively induced a wide range of genetic variability in two cowpea varieties, resulting in the selection of high-yielding mutant lines.

## Conclusions and Future Directions

The present study reflected the usefulness of quantitative evaluation of phenotypic markers for assessing induced genetic variability in cowpea varieties. Mutagens significantly increased the genetic parameters in the selected mutagenized populations and confirmed the scope of yield improvement in cowpea varieties. The data on quantitative traits indicated that mutagens were effective in inducing substantial inter-population genetic divergence. The increased mean values of quantitative traits in the mutagenized populations revealed a wide possibility of selecting yield trait (s). Yield based statistics in M_2_ generation revealed six superior high yielding mutagenized populations. In the M_3_ generation, two treatment lines, viz. G1 in variety Gomati VU-89 and S1 in variety Pusa-578 showed maximum genetic gain and could be advanced to subsequent generations at multiple locations. Correlation and path analysis showed that seed weight and seeds per pod are the main yield components and might be prioritized over other traits in indirect selection for yield trait (s). The HCA grouped mutant lines in different clusters with wide inter-cluster distance, indicating that mutagenic treatments induced heterogeneous populations in the two parental lines. PCA revealed that mutants treated with lower and higher mutagen doses were divergent and could be suitable in the crop improvement programs aimed at broadening the genetic base of cowpea. In the M_4_ generation, high yielding mutant lines showed improved genetic gain and increased nutrient density. Considering the improved genetic gain and nutrient density in selected mutant lines, further characterization and multi-location trails can be fruitful for releasing high yielding biofortified cowpea varieties. Besides, these high yielding biofortified mutant lines can serve as parents in crossbreeding programs to broaden the genetic base of cowpea.

## Data Availability Statement

The original contributions presented in the study are included in the article/Supplementary Material, further inquiries can be directed to the corresponding author/s.

## Author Contributions

AR contributed to designing and performing the experiments, analyzing and assessing data, and drafting and revising the manuscript. RL, MRW, and SA analyzed and interpreted data. BJ contributed in data analysis and funding acquisition. SK contributed to the supervision of overall experimentation. All authors reviewed the manuscript. All authors contributed to the article and approved the submitted version.

## Conflict of Interest

The authors declare that the research was conducted in the absence of any commercial or financial relationships that could be construed as a potential conflict of interest.

## Publisher's Note

All claims expressed in this article are solely those of the authors and do not necessarily represent those of their affiliated organizations, or those of the publisher, the editors and the reviewers. Any product that may be evaluated in this article, or claim that may be made by its manufacturer, is not guaranteed or endorsed by the publisher.

## Footnotes

^1^https://mvd.iaea.org/ (accessed April 27, 2022).
