# Supplementary material for: Gamma Rays and Sodium Azide Induced Genetic Variability in High-Yielding and Biofortified Mutant Lines in Cowpea [Vigna unguiculata (L.) Walp.]
Source: Front Plant Sci. 2022 Jun 14;13:911049. doi: 10.3389/fpls.2022.911049 (PMC9237497; doi:10.3389/fpls.2022.911049)

**Frontiers in Plant Science**

**Section Plant Breeding**

**Supplementary material**

**Gamma rays and sodium azide induced genetic variability in high yielding and biofortified mutant lines in cowpea [*Vigna unguiculata* (L.) Walp.]**

*Aamir Raina^1,2*^, Rafiul Amin Laskar^3^, Mohammad Rafiq Wani^4^, Basit Latief Jan^5^, Sajad Ali^6^ Samiullah Khan^1^*

^1^Mutation Breeding Laboratory, Department of Botany, Aligarh Muslim University, Aligarh, India

^2^Botany Section, Women’s College, Aligarh Muslim University, Aligarh, India

^4^Department of Botany, Abdul Ahad Azad Memorial Degree College Bemina, Cluster University Srinagar, Jammu and Kashmir, India

^3^Department of Botany, Bahona College, Jorhat, Assam, India

^5^Department of Clinical Pharmacy, College of Pharmacy, King Saud University, Riyadh, Saudi Arabia

^6^Department of Biotechnology, Yeungnam University, Republic of Korea

Correspondence

Dr. Aamir Raina

aamir854@gmail.com

**Table 1** Description of selected cowpea varieties.

|  | **Pedigree** | **Year / Place of release** | **Distinguishing characters** |
| --- | --- | --- | --- |
| **Variety Gomati VU-89** | Selection from local germplasm at Kanke | 1974/  RAU, Kanke, Ranchi | Maturity 150-155 days; Avg. yield 25-30 Q/ha; Resistant to yellow mosaic virus; 13.00 g/100 seeds; Long pods; Avg. 60 pods/plant; small seed size |
| **Variety Pusa-578** | Selection from  cv. EC170578 | 2005/  IARI, New Dehli | Maturity 155-160 days; Avg. yield 5 Q/ha; Resistant to yellow mosaic virus; 21.00 g/100 seeds; Short pods; Avg. 40 pods/plant; Large seed size |

**Table** **2** Description of mutant lines grown/selected in each generation from M_1_ to M_4_ in the variety Gomati VU-89.

| **Code** | **Mutagen** | **M_1_** | | | **M_2_** | | | | **M_3_** | | **M_4_** |
| --- | --- | --- | --- | --- | --- | --- | --- | --- | --- | --- | --- |
|  |  | **Seed Treated** | **Seeds Germinated** | **Fertile Plants** | **M_2_ Lines** | **Total M_2_ Seeds** | **Seeds Germinated** | **Fertile Plants** | **M_3_ Lines** | **Total M_3_ Plants** | **M_4_ High Yielding Lines** |
| C | **Control*** | 0 | 278 | 276 | 276 | 2760 | 2576 | 2378 | 0 | 0 | ns |
| G1 | **100 Gy γ rays** | 300 | 261 | 260 | 260 | 2600 | 2297 | 2013 | 30 | 300 | **2** |
| G2 | **200 Gy γ rays** | 300 | 255 | 254 | 254 | 2540 | 2184 | 1864 | 30 | 300 | **1** |
| G3 | **300 Gy γ rays** | 300 | 245 | 243 | 243 | 2430 | 2009 | 1634 | ns | ns | ns |
| G4 | **400 Gy γ rays** | 300 | 240 | 238 | 238 | 2380 | 1920 | 1542 | ns | ns | ns |
| S1 | **0.01% SA** | 300 | 260 | 259 | 259 | 2590 | 2262 | 1975 | 30 | 300 | **-** |
| S2 | **0.02% SA** | 300 | 254 | 252 | 252 | 2520 | 2167 | 1835 | 30 | 300 | **1** |
| S3 | **0.03% SA** | 300 | 246 | 243 | 243 | 2430 | 2025 | 1647 | ns | ns | ns |
| S4 | **0.04% SA** | 300 | 238 | 233 | 233 | 2330 | 1895 | 1478 | ns | ns | ns |
| G1+S1 | **100 Gy γ rays+0.01% SA** | 300 | 257 | 255 | 255 | 2550 | 2219 | 1893 | 30 | 300 | **1** |
| G2+S2 | **200 Gy γ rays+0.02% SA** | 300 | 245 | 243 | 243 | 2430 | 2033 | 1660 | 30 | 300 | **2** |
| G3+S3 | **300 Gy γ rays+0.03% SA** | 300 | 235 | 230 | 230 | 2300 | 1848 | 1429 | ns | ns | ns |
| G4+S4 | **400 Gy γ rays+0.04% SA** | 300 | 228 | 225 | 225 | 2250 | 1748 | 1316 | ns | ns | ns |
| T | **Total** | **3600** | **2964** | **2935** | **2935** | **29350** | **24607** | **20287** | **180** | **1800** | **7** |

*For control set, 300 seeds were sown in the same field. ns: no selection.

**Table** **3** Description of mutant lines grown/selected in each generation from M_1_ to M_4_ in the variety Pusa-578.

| **Code** | **Mutagen** | **M_1_** | | | **M_2_** | | | | **M_3_** | | **M_4_** |
| --- | --- | --- | --- | --- | --- | --- | --- | --- | --- | --- | --- |
|  |  | **Seed Treated** | **Seeds Germinated** | **Fertile Plants** | **M_2_ Lines** | **Total M_2_ Seeds** | **Seeds Germinated** | **Fertile Plants** | **M_3_ Lines** | **Total M_3_ Plants** | **M_4_ High Yielding Lines** |
| C | **Control*** | 0 | 272 | 270 | 270 | 2700 | 2484 | 2269 | 0 | 0 | ns |
| G1 | **100 Gy γ rays** | 300 | 251 | 250 | 250 | 2500 | 2125 | 1792 | 30 | 300 | ns |
| G2 | **200 Gy γ rays** | 300 | 245 | 244 | 244 | 2440 | 2033 | 1660 | 30 | 300 | **1** |
| G3 | **300 Gy γ rays** | 300 | 230 | 238 | 238 | 2380 | 1944 | 1562 | ns | ns | ns |
| G4 | **400 Gy γ rays** | 300 | 220 | 233 | 233 | 2330 | 1872 | 1466 | ns | ns | ns |
| S1 | **0.01% SA** | 300 | 250 | 258 | 258 | 2580 | 2279 | 1983 | 30 | 300 | ns |
| S2 | **0.02% SA** | 300 | 244 | 242 | 242 | 2420 | 2033 | 1653 | 30 | 300 | **1** |
| S3 | **0.03% SA** | 300 | 229 | 233 | 233 | 2330 | 1856 | 1460 | ns | ns | ns |
| S4 | **0.04% SA** | 300 | 219 | 226 | 226 | 2260 | 1725 | 1317 | ns | ns | ns |
| G1+S1 | **100 Gy γ rays+0.01% SA** | 300 | 242 | 245 | 245 | 2450 | 2050 | 1688 | 30 | 300 | **1** |
| G2+S2 | **200 Gy γ rays+0.02% SA** | 300 | 225 | 232 | 232 | 2320 | 1872 | 1454 | 30 | 300 | **1** |
| G3+S3 | **300 Gy γ rays+0.03% SA** | 300 | 210 | 224 | 224 | 2240 | 1732 | 1311 | ns | ns | ns |
| G4+S4 | **400 Gy γ rays+0.04% SA** | 300 | 205 | 202 | 202 | 2020 | 1522 | 1116 | ns | ns | ns |
| T | **Total** | **3600** | **2770** | **2827** | **2827** | **28270** | **23043** | **18462** | **180** | **1800** | **4** |

* For control set, 300 seeds were sown in the same field. ns: no selection

| **Sl. No.** | **Traits** | **Method of measurement** |
| --- | --- | --- |
| 1 | Plant height (cm) | height from the base up to the apex of the plant |
| 2 | Days to flowering | days are taken from sowing to the date of opening of the first flower |
| 3 | Days to maturity | days are taken from sowing until the date of harvesting |
| 4 | Pods per plant (number) | total number of pods per plant |
| 5 | Branches per plant (number) | number of branches per plant at maturity |
| 6 | Seeds per pod (number) | number of seeds in a pod |
| 7 | Seed weight (g) | weight of a random sample of hundred seeds |
| 8 | Pod length (cm) | pod length was measured from the base to the tip of the pod |
| 9 | Plant yield (g) | weight of the total number of seeds harvested |
| 10 | Harvest Index (%) | the ratio of grain yield to biological yield |

**Table 4.** List of phenotypic traits and description of their quantitative measurements.

| Treatments | **Variety Gomati VU-89** | | | | | **Variety Pusa-578** | | | | |
| --- | --- | --- | --- | --- | --- | --- | --- | --- | --- | --- |
|  | M_2_ | | M_3_ | | Overall Genetic Gain  (%) | M_2_ | | M_3_ | | Overall Genetic Gain  (%) |
|  | Mean (g) | Genetic Gain  (%) | Mean(g) | Genetic Gain  (%) |  | Mean(g) | Genetic Gain  (%) | Mean(g) | Genetic Gain  (%) |  |
| **C** | 93.75 | ----- | 92.64 | ----- | ----- | 83.51 | ----- | 80.29 | ----- | ----- |
| **G1** | 112 | 19.47 | 119.27 | 28.75 | 9.28 | 105.54 | 26.38 | 112.83 | 40.53 | 14.15 |
| **G2** | 106.67 | 13.78 | 110.52 | 19.30 | 5.52 | 101.9 | 22.02 | 108.42 | 35.04 | 13.01 |
| **G3** | 102.28 | 9.10 | ----- | ----- | ----- | 93.17 | 11.57 | ----- | ----- | ----- |
| **G4** | 97.42 | 3.91 | ----- | ----- | ----- | 86.09 | 3.09 | ----- | ----- | ----- |
| **S1** | 110.01 | 17.34 | 115.28 | 24.44 | 7.09 | 110.63 | 32.48 | 117.01 | 45.73 | 13.26 |
| **S2** | 108 | 15.20 | 112.9 | 21.87 | 6.67 | 97.76 | 17.06 | 101.25 | 26.11 | 9.04 |
| **S3** | 105.5 | 12.53 | ----- | ----- | ----- | 94.09 | 12.67 | ----- | ----- | ----- |
| **S4** | 95.94 | 2.34 | ----- | ----- | ----- | 81.01 | -2.99 | ----- | ----- | ----- |
| **G1+S1** | 103.8 | 10.72 | 106.72 | 15.20 | 4.48 | 100.38 | 20.20 | 105.9 | 31.90 | 11.70 |
| **G2+S2** | 106.97 | 14.10 | 109.99 | 18.73 | 4.63 | 89.37 | 7.02 | 94.8 | 18.07 | 11.05 |
| **G3+S3** | 90.52 | -3.45 | ----- | ----- | ----- | 83.29 | -0.26 | ----- | ----- | ----- |
| **G4+S4** | 87.8 | -6.35 | ----- | ----- | ----- | 78.39 | -6.13 | ----- | ----- | ----- |

**Table 5** Genetic gain in plant yield from M_2_ and M_3_ generation and overall gain from M_2_ to M_3_ generation in Cowpea varieties Gomati VU-89

C = Control; G1 = 100 Gy γ rays; G2 = 200 Gy γ rays; G3 = 300 Gy γ rays; G4 = 400 Gy γ rays; S1 = 0.01% SA; S2 = 0.02% SA; S3 = 0.03% SA; S4 = 0.04% SA; G1+S1 = 100 Gy γ rays+0.01% SA; G2+S2 = 200 Gy γ rays+0.02% SA; G3+S3 = 300 Gy γ rays+0.03% SA; G4+S4 = 400 Gy γ rays+0.04% SA.and Pusa-578.

**Table 6** One-Way ANOVA results for the ten phenotypic quantitative traits of cowpea varieties in M_2_ generation. Significant difference are indicated as ‘***’ for P < 0.001, ‘**’ for P < 0.01, ‘*’ for P < 0.05, ‘**.**’ for P < 0.1 and ‘ns’ for non significance, df represent degree of freedom.

| **Traits** | **Source of Variation**  **(Treatments)** | **df** | **Gomati VU-89** | | | **Pusa-578** | | |
| --- | --- | --- | --- | --- | --- | --- | --- | --- |
|  |  |  | **Mean Square** | **F** | **p** | **Mean Square** | **F** | **p** |
| Plant height (cm) | Between | 12 | 186.2 | 60.02 | 0.00^**^ | 107.99 | 8.97 | 0.00^**^ |
|  | Within | 377 | 3.1 |  |  | 12.03 |  |  |
| Days to flowering | Between | 12 | 147.06 | 9.53 | 0.00^**^ | 66.70 | 9.63 | 0.00^**^ |
|  | Within | 377 | 15.42 |  |  | 6.92 |  |  |
| Days to maturity | Between | 12 | 238.28 | 20.52 | 0.00^**^ | 163.18 | 11.92 | 0.00^**^ |
|  | Within | 377 | 11.61 |  |  | 13.69 |  |  |
| Pods per  Plant | Between | 12 | 345.2 | 21.27 | 0.00^**^ | 200.77 | 27.43 | 0.00^**^ |
|  | Within | 377 | 16.2 |  |  | 7.32 |  |  |
| Branches per  plant | Between | 12 | 38.04 | 28.28 | 0.00^**^ | 47.31 | 29.46 | 0.00^**^ |
|  | Within | 377 | 1.34 |  |  | 1.61 |  |  |
| Seeds per  Pod | Between | 12 | 1.68 | 1.57 | 0.09^ns^ | 1.79 | 1.68 | 0.11^ns^ |
|  | Within | 377 | 1.07 |  |  | 1.12 |  |  |
| 100 seed weight (g) | Between | 12 | 1.51 | 1.55 | 0.10^ns^ | 9.27 | 3.54 | 0.00^**^ |
|  | Within | 377 | 0.97 |  |  | 2.61 |  |  |
| Pod length  (cm) | Between | 12 | 22.92 | 27.23 | 0.00^**^ | 48.44 | 32.96 | 0.00^**^ |
|  | Within | 377 | 0.84 |  |  | 1.47 |  |  |
| Plant yield  (g) | Between | 12 | 1798.6 | 108.9 | 0.00^**^ | 3068.1 | 412 | 0.00^**^ |
|  | Within | 377 | 16.5 |  |  | 7.4 |  |  |
| Harvest index per plant (%) | Between | 12 | 514.07 | 109.6 | 0.00^**^ | 328.9 | 88.44 | 0.00^**^ |
|  | Within | 377 | 4.70 |  |  | 3.7 |  |  |

**Table 7:** Correlation coefficient matrix for yield and photopigments, enzymes, proteins, and micronutrients in M_4_ high yielding mutants.

| **Mutants** | **Characters** | **Chlorophyll** | **Carotenoids** | **Nitrate reductase activity** | **Protein** | **Iron** | **Zinc** | **Copper** |
| --- | --- | --- | --- | --- | --- | --- | --- | --- |
| **Gomati VU-89** | **Yield** | **-0.631** | **0.251** | **0.531** | **+0.153** | **+0.525** | **+0.172** | **+0.277** |
| Gomati VU-89-A |  | **+0.942** | **+0.984** | **-0.805** | **+0.013** | +0.997 | +0.934 | +0.972 |
| Gomati VU-89-B |  | +0.934 | +1.000 | +0.802 | +0.992 | +0.941 | +0.998 | -0.447 |
| Gomati VU-89-C |  | +0.931 | +1.000 | +0.163 | +0.999 | +0.909 | +0.127 | +0.923 |
| Gomati VU-89-D |  | +0.805 | +0.837 | +0.947 | +0.679 | +0.592 | +0.208 | +0.930 |
| Gomati VU-89-E |  | +0.936 | +0.989 | -0.916 | +0.719 | +0.669 | +0.629 | +0.989 |
| Gomati VU-89-F |  | +0.997 | +0.991 | +0.114 | +0.857 | +0.992 | +0.935 | +0.691 |
| Gomati VU-89-G |  | +0.898 | +0.984 | +0.789 | +0.1000* | +0.891 | +0.832 | +0.907 |
| **Pusa-578** |  | **+0.811** | **+0.976** | **+0.787** | **+0.895** | **+0.765** | **-0.744** | **-0.832** |
| Pusa-578-A |  | +0.595 | +0.387 | +0.595 | +0.923 | -0.560 | -0.985 | -0.894 |
| Pusa-578-B |  | +0.974 | +0.972 | +0.994 | +0.833 | -0.335 | -0.752 | -0.947 |
| Pusa-578-C |  | +0.823 | +0.981 | **+1.000**** | +0.960 | +0.876 | +0.836 | +0.990 |
| Pusa-578-D |  | **+1.000*** | **+0.976** | **+0.927** | **+1.000*** | -0.749 | +0.212 | +0.864 |

**Table 8.** Characteristic means of three cluster groups of thirteen populations of cowpea varieties Gomati VU-89 and Pusa-578.

| **Characters** | **var. Gomati VU-89** | | | **var. Pusa-578** | | |
| --- | --- | --- | --- | --- | --- | --- |
|  | **Cluster 1** | **Cluster 2** | **Cluster 3** | **Cluster 1** | **Cluster 2** | **Cluster 3** |
| **Plant Height** | 182.62 | 180.01 | 174.37 | 173.3 | 179.3 | 178.11 |
| **Days to Flowering** | 80.17 | 77.67 | 78.53 | 84.7 | 84.63 | 86.23 |
| **Days to Maturity** | 154.83 | 150.23 | 143.13 | 150.1 | 156.1 | 156 |
| **Pods Per Plant** | 60.10 | 65.97 | 56.97 | 38.27 | 44.9 | 43.8 |
| **Branches Per Plant** | 8.63 | 11.35 | 9.77 | 11.1 | 13.7 | 13.23 |
| **Seeds Per Pod** | 12.00 | 12.47 | 11.97 | 9.6 | 11 | 10.1 |
| **Seed Weight** | 13.00 | 13.49 | 12.90 | 21.35 | 22.4 | 22.1 |
| **Pod Length** | 29.59 | 30.71 | 29.18 | 24.09 | 26.72 | 25.88 |
| **Plant Yield** | 93.75 | **111.37** | 87.80 | 78.39 | **110.64** | 97.77 |
| **Harvest Index** | 27.14 | **40.84** | 30.06 | 27.75 | **37.54** | 30.53 |

**Table 9.** Intercluster distances among populations of cowpea varieties Gomati VU-89 and Pusa-578.

| **Variety Gomati VU-89** |  | CLUSTER I | CLUSTER II | CLUSTER III |  | **Variety Pusa-578** |
| --- | --- | --- | --- | --- | --- | --- |
|  | CLUSTER I | **** | 25.45 | 13.60 | CLUSTER I |  |
|  | CLUSTER II | 19.25 | **** | 16.81 | CLUSTER II |  |
|  | CLUSTER III | 12.75 | 11.91 | **** | CLUSTER III |  |

| **Variety Gomati** **VU-89** |  | **C** | **G1** | **G2** | **G3** | **G4** | **S1** | **S2** | **S3** | **S4** | **G1+S1** | **G2+S2** | **G3+S3** | **G4+S4** | **Variety Pusa-578** |
| --- | --- | --- | --- | --- | --- | --- | --- | --- | --- | --- | --- | --- | --- | --- | --- |
|  | **C** |  | 24.27 | 23.48 | 13.38 | 9.55 | **29.39** | 15.59 | 13.82 | 8.23 | 20.00 | 11.40 | 10.33 | 14.09 |  |
|  | **G1** | **24.00** |  | 7.58 | 12.62 | 20.19 | 6.54 | 9.73 | 11.64 | 25.07 | 6.07 | 16.79 | 23.90 | 29.77 |  |
|  | **G2** | 19.60 | 5.49 |  | 11.65 | 19.21 | 10.22 | 10.79 | 10.53 | 23.41 | 6.17 | 14.76 | 22.96 | 28.98 |  |
|  | **G3** | 15.87 | 10.44 | 5.01 |  | 8.00 | 18.37 | 6.80 | ***1.92*** | 12.68 | 8.07 | 4.88 | 12.04 | 18.11 |  |
|  | **G4** | 12.94 | 15.96 | 10.57 | 5.75 |  | 25.93 | 12.98 | 9.45 | 5.59 | 15.41 | 5.53 | 5.04 | 10.61 |  |
|  | **S1** | 22.27 | 4.00 | 5.45 | 9.49 | 14.86 |  | 14.89 | 17.15 | 30.54 | 10.94 | 22.10 | 29.71 | **35.62** |  |
|  | **S2** | 20.74 | 4.59 | 2.47 | 6.75 | 12.13 | 5.77 |  | 6.23 | 17.51 | 6.23 | 9.72 | 15.97 | 21.97 |  |
|  | **S3** | 17.54 | 8.00 | 3.78 | 4.72 | 9.21 | 8.55 | 4.20 |  | 13.74 | 7.17 | 5.91 | 13.58 | 19.69 |  |
|  | **S4** | 12.88 | 18.89 | 13.58 | 9.01 | 3.73 | 17.90 | 15.08 | 11.86 |  | 20.11 | 9.25 | 5.98 | 9.17 |  |
|  | **G1+S1** | 16.41 | 8.77 | 3.86 | 3.10 | 7.68 | 8.40 | 4.84 | ***2.66*** | 10.79 |  | 11.47 | 19.06 | 25.01 |  |
|  | **G2+S2** | 19.76 | 7.63 | 5.81 | 8.04 | 12.11 | 9.16 | 4.63 | 4.19 | 14.73 | 5.27 |  | 8.80 | 14.83 |  |
|  | **G3+S3** | 13.68 | 24.91 | 19.76 | 15.28 | 9.91 | 23.74 | 20.91 | 18.08 | 7.68 | 16.59 | 20.26 |  | 6.22 |  |
|  | **G4+S4** | 16.21 | **29.00** | 23.98 | 19.60 | 14.21 | 27.89 | 25.03 | 22.14 | 11.65 | 20.76 | 24.02 | 4.64 |  |  |

**Table 10.** Interpopulation proximity matrix based on Euclidean distance in the different populations of cowpea varieties Gomati VU-89 and Pusa-578.

**Figure 1:** Comparative effects of gamma radiations on M_2_ plant survival (%) in cowpea varieties Gomati VU-89 and Pusa-578.

C = Control; G1 = 100 Gy γ rays; G2 = 200 Gy γ rays; G3 = 300 Gy γ rays; G4 = 400 Gy γ rays

**Figure 2:** Comparative effects of sodium azide on M_2_ plant survival (%) in cowpea varieties Gomati VU-89 and Pusa-578.

C = Control; S1 = 0.01% SA; S2 = 0.02% SA; S3 = 0.03% SA; S4= 0.04% SA;

**Figure S3:** Comparative effects of gamma raditions+sodium azide on M_2_ plant survival (%) in cowpea varieties Gomati VU-89 and Pusa-578.

C = Control; G1+S1= 100 Gy γ rays+0.01% SA; G2+S2 = 200 Gy γ rays+0.02% SA; G3+S3 = 300 Gy γ rays+0.03% SA; G4+S4 = 400 Gy γ rays+0.04% SA.

**Figure 4:** Field plots layout of M_1_ generations in a randomized complete block design (RCBD) for each cultivar. (Source: Raina et al., 2020. http://creativecommons.org/licenses/by/4.0/).
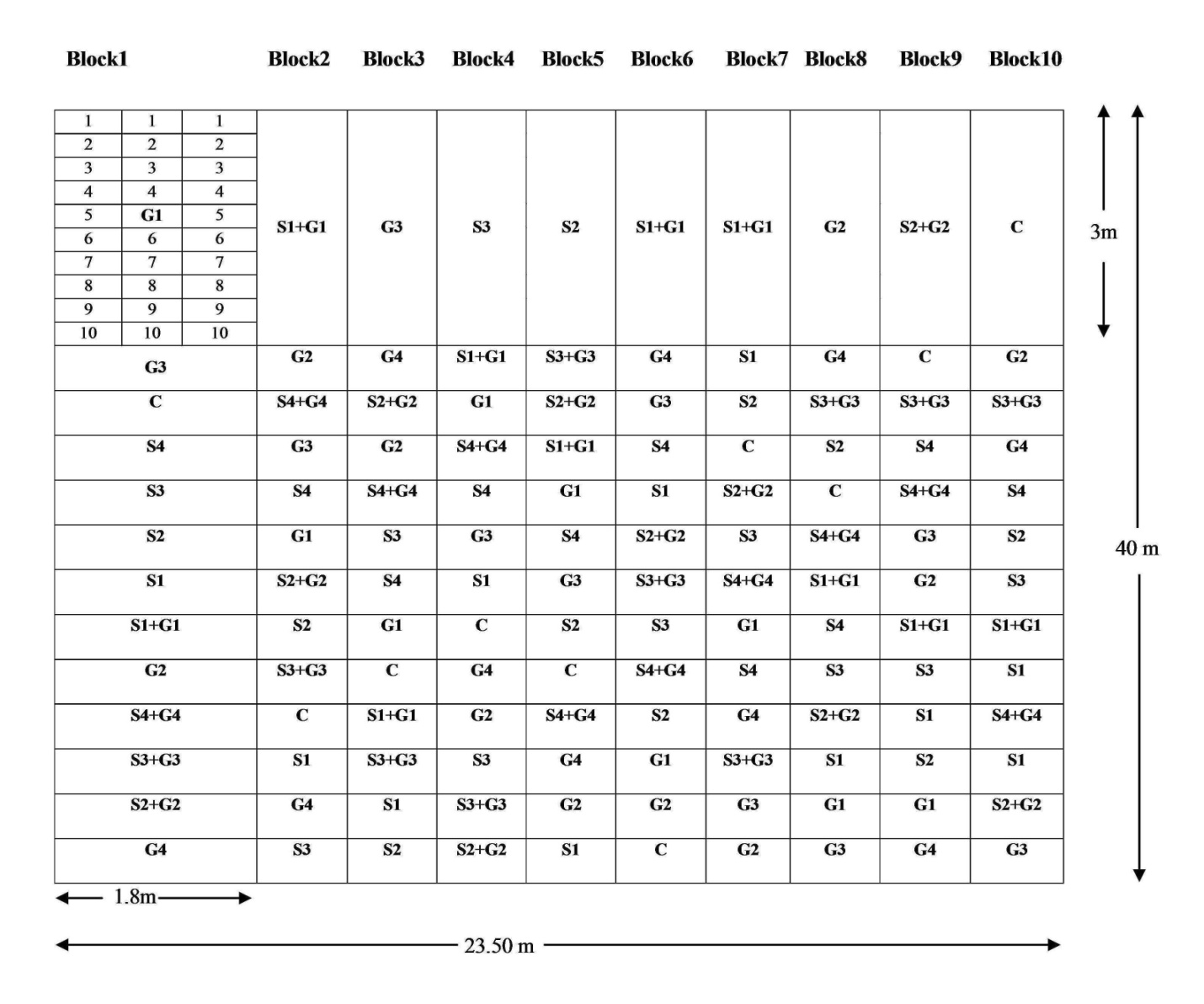

Supplement: Supplementary file 1 [file Table_1.DOCX]
